# Puncturing lipid membranes: onset of pore formation and the role of hydrogen bonding in the presence of flavonoids

**DOI:** 10.1016/j.jlr.2023.100430

**Published:** 2023-08-22

**Authors:** Anja Sadžak, Zlatko Brkljača, Mihael Eraković, Manfred Kriechbaum, Nadica Maltar-Strmečki, Jan Přibyl, Suzana Šegota

**Affiliations:** 1 Division of Physical Chemistry, Ruđer Bošković Institute, Zagreb, Croatia; 2 Division of Organic Chemistry and Biochemistry, Ruđer Bošković Institute, Zagreb, Croatia; 3 Institute of Inorganic Chemistry, Graz University of Technology, Graz, Austria; 4 CEITEC MU, Masaryk University, Brno, Czech Republic

**Keywords:** antioxidant, dicarboxylic acid, flavone, flavonol, lipid/peroxidation, oxidized lipid, phospholipid/phosphatidylcholine, physical biochemistry

## Abstract

Products of lipid peroxidation induce detrimental structural changes in cell membranes, such as the formation of water pores, which occur in the presence of lipids with partially oxidized chains. However, the influence of another class of products, dicarboxylic acids, is still unclear. These products have greater mobility in the lipid bilayer, which enables their aggregation and the formation of favorable sites for the appearance of pores. Therefore, dodecanedioic acid (DDA) was selected as a model product. Additionally, the influence of several structurally different flavonoids on DDA aggregation via formation of hydrogen bonds with carboxyl groups was investigated. The molecular dynamics of DDA in DOPC lipid bilayer revealed the formation of aggregates extending over the hydrophobic region of the bilayer and increasing its polarity. Consequently, water penetration and the appearance of water wires was observed, representing a new step in the mechanism of pore formation. Furthermore, DDA molecules were found to interact with lipid polar groups, causing them to be buried in the bilayer. The addition of flavonoids to the system disrupted aggregate formation, resulting in the displacement of DDA molecules from the center of the bilayer. The placement of DDA and flavonoids in the lipid bilayer was confirmed by small-angle X-ray scattering. Atomic force microscopy and electron paramagnetic resonance were used to characterize the structural properties. The presence of DDA increased bilayer roughness and decreased the ordering of lipid chains, confirming its detrimental effects on the membrane surface, while flavonoids were found to reduce or reverse these changes.

## Graphical abstract

**Figure:**
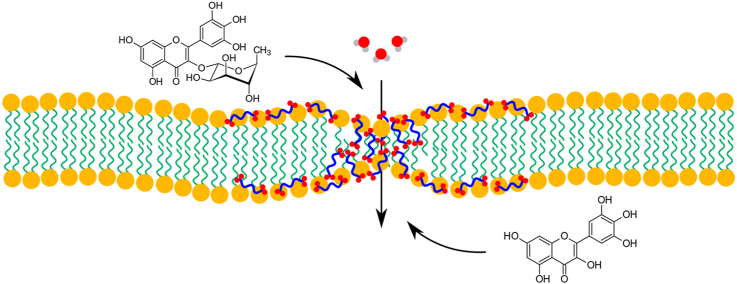

The cell membrane, composed of lipids and proteins, separates the intracellular from the extracellular space and regulates the transport of substances in and out of the cell. All membrane lipids are amphiphilic and in aqueous environment form a lipid bilayer, which is the structural core of all biomembranes (1). The predominant type of lipids in cell membranes are phospholipids, containing two fatty acids attached to the 1,2-hydroxyl groups of glycerol (2, 3). Many of these acids are polyunsaturated, with the double bonds separated by reactive methylene bridges. The dissociation energies of bisallylic carbon-hydrogen bonds are low, allowing facile elimination of hydrogen atoms by radical species and subsequent reaction with molecular oxygen (4, 5). The high concentration of polyunsaturated fatty acids (PUFAs) makes membranes susceptible to oxidation and enables their participation in radical chain reactions, e.g., lipid peroxidation. Lipid peroxidation is a process caused by reactive oxygen species that leads to the degradation of lipids (6, 7). It occurs under conditions of oxidative stress and results in cell damage (8). In addition to the formation of toxic products (7, 9), which have been associated with alteration of signal transduction pathways (6), modification of enzyme activities (10, 11), and membrane protein function (12, 13), lipid peroxidation causes structural damage to target systems (3, 14, 15) and impairs membrane dynamics and barrier function. Oxidized species in the lipid membrane can stimulate cell signaling and phase separation with the appearance of lipid domains (16, 17). All of these effects contribute to oxidative stress being associated with various diseases, including neurodegenerative diseases (18, 19, 20), as well as disorders of gastrointestinal tract (21), kidney, and liver (22).

There are a number of initiation mechanisms used to study lipid peroxidation, eg, the Fenton reaction (23, 24) and UV irradiation (25), where batch oxidation of phospholipids occurs and effects on the bilayer structure are attributed to a wide range of oxidation products. However, these changes differ when the impact of well-defined products is investigated. In fact, Megli and Russo (26) reported that the use of specific lipid oxides allows the detection of changes that are not detectable when oxidation mixtures are used. Therefore, to understand the complexity of the influence of lipid peroxidation on membrane integrity, it is crucial to study individual oxidation products. The effect of structurally defined oxidized lipids on the membrane structure has been studied using model phospholipid membranes, which has allowed exploration of the physical effects of oxidation (27, 28). One of the most important structural effects induced by lipid peroxidation is an increase in water permeability and a decrease in bilayer thickness. The decrease in bilayer thickness is a consequence of partial interdigitation of terminal methyl segments of acyl chains (29, 30). In addition, changes in lipid membrane order and fluidity, as well as the appearance of various defects, have been reported (25, 31). In the case of pronounced defects, disintegration of the membrane structure and subsequent pore formation occur. For example, Cwiklik and Jungwirth (32) studied structural changes in the 1,2-dioleoyl-sn-glycero-3-phosphocholine (DOPC) membrane after oxidizing one or both unsaturated acyl chains and delineated the disintegration of the bilayer and mechanism of pore formation. Briefly, the pore walls appear as a result of increased average distance between polar head groups caused by insertion of an oxidized chain into the polar region and water permeation through the membrane, followed by direct interaction of phosphate groups located on different sides of the bilayer (32). Formation of pores was observed in the cases of lecithin (33) and other phosphatidylcholine lipids (34, 35).

In most of the studies performed, investigated products were lipid molecules in which one or both chains were oxidized, studied in terms of their parent molecules (32, 35). However, lipid peroxidation is a complex reaction involving loss of double bonds, shortening of chains, and introduction of various compounds with terminal polar groups (1, 3, 23), among which are medium- and long-chain dicarboxylic acids (23, 36). Inouye *et al.* (37) reported formation of short- and medium-chain length dicarboxylic acids, such as suberic and adipic acid, during *cis*-PUFAs oxidation and proposed them as markers of oxidation in diabetes. The variety of dicarboxylic products was further confirmed by Passi *et al.* (38) who reported formation of two different dicarboxylic acids from a single fatty acid, one deriving from the oxidative splitting at the level of the first double bond in the molecule and the other being two-carbon-atoms lower homologous. Because of their multiple carboxyl groups, the interaction with the lipid bilayer differs from structural damage caused by compounds with only one carbonyl or carboxyl group (26). In particular, dicarboxylic acids that interact with polar groups via hydrogen bonds exhibit greater mobility compared to products with oxidized chains covalently bonded to the polar head group. Consequently, the mechanism of structural changes induced by this class of products is expected to be significantly different. In our research, we used dodecanedioic acid (DDA), which belongs to the sequence of saturated dicarboxylic acids already identified as peroxidation products (23, 24, 36, 37, 38, 39).This molecule was chosen to understand the behavior of lipid bilayer in the presence of peroxidation products that can form interactions, primarily hydrogen bonds, on both sides of the hydrocarbon chain.

Flavonoids are naturally occurring polyphenolic compounds that exhibit antioxidant behavior by either directly scavenging radicals or by forming chelate complexes with metal ions, which are known to catalyze lipid peroxidation (40, 41, 42). The interaction of flavonoids with lipid bilayers is widely studied because of their protective behavior during oxidative stress (43, 44, 45). Depending on structural properties, ie, hydrophobicity, flavonoids position themselves differently within the bilayer (46, 47, 48). Like lipids, flavonoids are amphipathic molecules, in which the hydroxyl groups are hydrophilic and the aromatic rings are hydrophobic (49). Consequently, the position and orientation of polyphenols in the membrane depend on the number and position of hydroxyl groups (50). Furthermore, the flavonoids in their glycoside form have additional polar sugar moieties, which further influences their positioning inside the bilayer. Some flavonoids have been shown to form clusters or aggregates inside lipid bilayer at sufficiently high concentrations (46, 51, 52). The clustering of flavonoids leads to subtle changes in the properties of the bilayer, which may affect its function (51). Because flavonoids have hydroxyl groups that can form hydrogen bonds, they can interact with products of lipid peroxidation and affect the structural changes of the membrane. Disruption of the interaction of oxidation products with polar heads may result in less pronounced structural changes and preservation of membrane integrity upon lipid peroxidation (24).

To study the interactions of a lipid bilayer with specific lipid peroxidation product, we prepared liposomes composed of DOPC lipid containing a prototypical product, DDA. A multitechnique approach, combining molecular dynamics (MD) simulation and experimental techniques, in particular, small-angle X-ray scattering (SAXS), electron paramagnetic resonance (EPR), and atomic force microscopy (AFM), was used. In addition, we studied incorporation of specific flavonoids into lipid bilayer to gain better insight into the interactions between antioxidants and lipid peroxidation products and to evaluate the ability of flavonoids to maintain the integrity of the bilayer. Because the interaction of flavonoids with lipid molecules in bilayers occurs mainly via hydrogen bonds (53), aglycone and glycone forms of the same flavonoid, namely, myricetin (MCE) and myricitrin (MCI), were studied. That way, subtle differences observed in the study of interactions could be attributed to specific substituents of the flavonoids. Furthermore, we studied flavonoids with different numbers of hydroxyl groups to investigate the dependence of bilayer properties on the degree of hydroxylation (Fig. 1). Quercetin (QUE) is a widely studied flavonol, which is abundant in fruits and vegetables, as well as in coffee and tea (54, 55, 56, 57, 58). In contrast to MCE and MCI, which have three hydroxyl groups on the B ring, QUE has two hydroxyl groups, indicating less pronounced antioxidant activity (59). In addition to studying compounds belonging to the flavonol subgroup, we also studied two flavones that do not possess hydroxyl group on the C ring. Apigenin (API) has been shown to inhibit inflammation, oxidation, and cancer cell growth (60). It has also been mentioned as a potential candidate for the treatment of dermatological disorders (61). Luteolin (LUT), whose health-promoting effects have already been confirmed in the literature, has an additional hydroxyl group compared with API. In particular, Zhu *et al.* (62) have shown that it reduces neuroinflammation-induced death in primary hippocampal neurons, while Seelinger *et al.* (63) have published a detailed review of its anticarcinogenic effects.

**Fig. 1:**
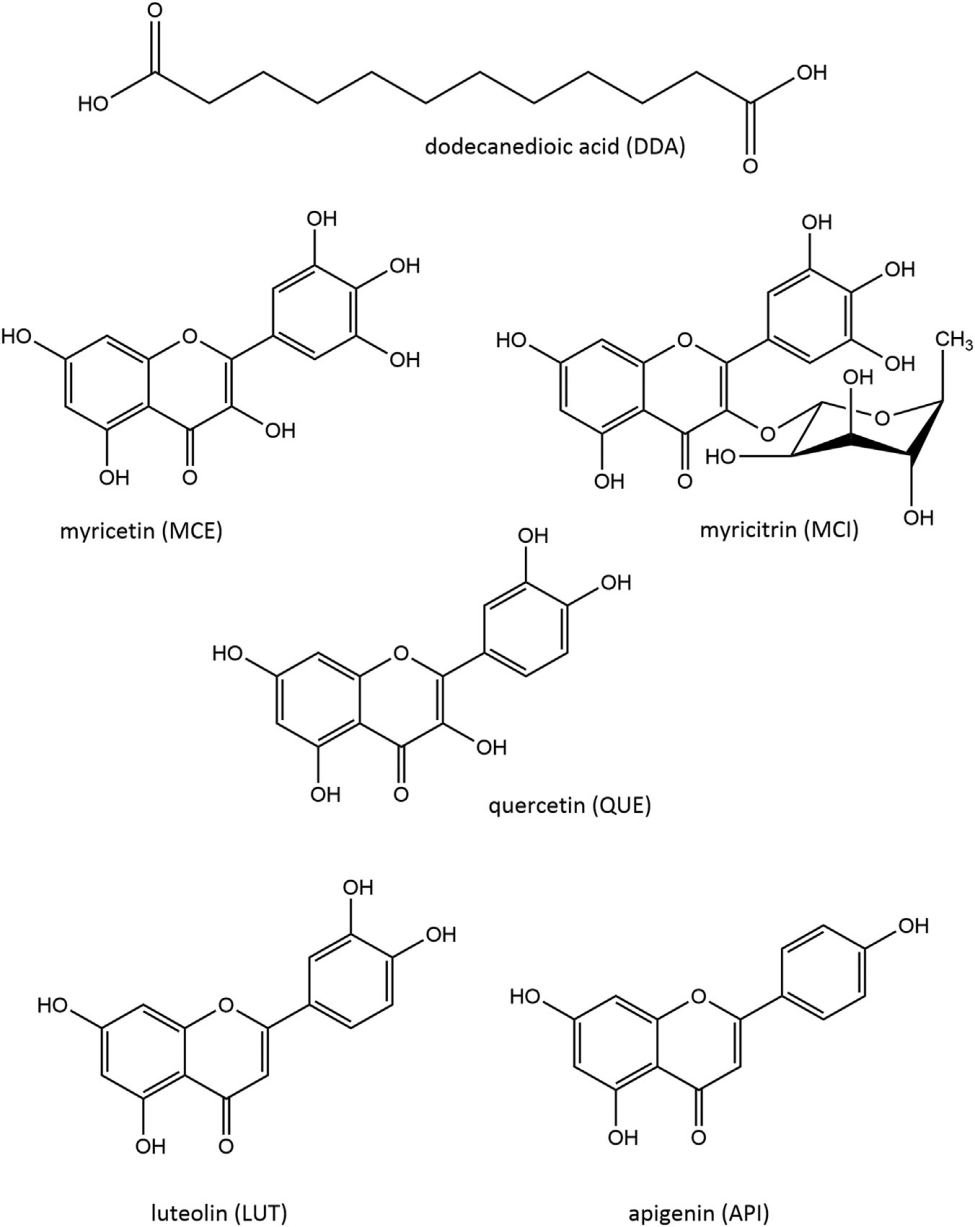
Structures of DDA and flavonoids used in this study.

In this paper, we elucidate the complex interplay of interactions between polar groups of lipid molecules, DDA, and flavonoids that leads to the alteration of membrane structural parameters. We present evidence for aggregation of DDA in the membrane leading to possible water pore formation. This mechanism of pore formation is different from the one proposed in the case of partially oxidized lipid molecules (POLMs). In addition, we show how the interaction of DDA with flavonoids reduces propensity for pore formation, indicating less pronounced damage caused by lipid peroxidation products and the protective role of flavonoids.

## Materials and Methods

### Chemicals

1,2-Dioleoyl-sn-glycero-3-phosphocholine (DOPC) was purchased from Avanti Lipids (Industrial Park Drive Alabaster, AL). Myricetin (MCE) (>97%) and myricitrin (MCI) (>98%) were purchased from TCI Chemicals Pvt. Ltd. (Chennai, India). Dodecanedioic acid (DDA) (99%), quercetin (QUE) (>95%), 5-doxyl-stearic acid (5-DSA), and phosphate-buffered saline (PBS) (PBS tablets, pH 7.4, *I*_*c*_ = 150 mM) were purchased from Sigma-Aldrich (St. Louis, MO). Luteolin (LUT) (97%) and apigenin (API) (97%) were purchased from Alfa Aesar (Haverhill, MA). Chloroform (99.93%) p.a. was purchased from Lach-ner Ltd. (Neratovice, Czech Republic). All chemicals were used without further purification.

### Preparation of liposomes

Liposomes were prepared by using the thin film hydration method, well described in the literature (64). Briefly, stock solutions (5 × 10^−4^ M) of DOPC, DDA, 5-DSA, and flavonoids were prepared by dissolving the solid compounds in chloroform and mixed in ratios corresponding to the desired molar fractions. Chloroform was evaporated using a rotary evaporator until the thin film appeared on the round flask, which was further dried in a vacuum. The dried films were hydrated using PBS solution, and the suspension was mixed at room temperature and immersed in ice and hot water to achieve scraping of film off the wall of the flask. All suspensions were stabilized overnight. The final concentration of liposomes was adjusted for each technique and is mentioned in the corresponding sections. Molar fractions of each flavonoid and DDA were 5% and 10%, respectively.

### Molecular dynamics simulations

To understand the influence of flavonoids on bilayers under oxidative stress, we performed a set of classical MD simulations. The simulated systems consist of either a pure DOPC bilayer (128 lipid units) or a DOPC bilayer in 1:1 ratio with a model product of lipid peroxidation, DDA. There are several reasons why the study of high oxidation degrees in model systems is important, as indicated by Van der Paal *et al.* (14). Since lipid peroxidation follows a chain reaction mechanism, products initially form close to each other. Furthermore, more polar compounds, ie, short-chain products, are known to aggregate in a lipophilic environment. Hence, a high concentration of products appears locally, in the hydrophobic center of the membrane. For each simulated membrane system, we performed three distinct simulations. More precisely, we simulated the bilayers without the presence of flavonoids (reference systems) and systems containing 13 flavonoid moieties (corresponding to approximately 5%–10% ratio of flavonoids to lipids). This molar fraction of flavonoids was chosen because it has been shown that at too high concentrations flavonoids exhibit prooxidant behavior (65). To parameterize DOPC moieties we utilized the Slipids force field (66, 67, 68, 69). Flavonoids and DDA were parameterized using GAFF55 (general Amber force field), consistently with the parameters used to describe lipids. Missing parameters, namely, partial charges, were obtained in a manner consistent with the general AMBER force field. More specifically, the partial charges for flavonoids were obtained from a restrained single-conformer fit to the electrostatic potential (RESP), respectively, while a 10-conformer RESP procedure was used to obtain partial charges of DDA, due to significantly more conformational freedom it possesses compared with the remaining parameterized species (67). The electrostatic potential was obtained from the HF/6-31G(d)//B3LYP/6-31G(d) quantum mechanical method. Each simulated system was solvated with 16,000 water molecules, whereby the standard TIP3P model was used to describe water molecules. Potassium chloride was added in all systems (67), with ionic strength set to 0.15 M, mirroring experimental conditions. The initial configurations of the simulated systems were prepared using Packmol (70), with DOPC lipids being oriented with their polar heads outward (toward water layer), with the nonpolar tails of two layers pointing toward each other, overall conferring with the common lipid bilayer setup. On the other hand, positions and orientations of DDA and flavonoid moieties were randomized, with them being set to initially lie inside the lipid bilayer (distance of the most protruding atom of each moiety set to lie at least 3 Å below/above the plane defined via the positions of polar lipid heads belonging to the upper/lower layer, respectively). All simulated systems were propagated for 200 ns. The simulations were performed in GROMACS 2018.4(67) with a time step of 2 fs, van der Waals and short-range Coulomb cutoffs of 12 Å, three-dimensional periodic boundary conditions, incorporating the particle mesh Ewald procedure. The systems were first minimized following short (10 ns) Langevin dynamics at *T* = 300 K, using Berendsen barostat (*p* = 1 bar) to relax the systems and obtain valid starting densities. The obtained systems were then propagated using the Nosé-Hoover thermostat at *T* = 300 K with Parrinello-Rahman barostat (*p* = 1 bar, semi-isotropic pressure coupling) for 200 ns, constituting the production runs. The subsequent analysis is performed considering the last 150 ns.

### Small-angle X-ray scattering

SAXS measurements were carried out on liposomes made of DOPC, DOPC + DDA, and DOPC + DDA with inserted MCE and MCI. These two flavonoids were chosen because of their greatest structural differences and the fact that other experimental and theoretical methods showed similar behavior among all studied flavonoids. The final concentration of liposomes for SAXS measurements was 50 mg mL^−1^. Electron density functions were obtained using a laboratory SAXS instrument (SAXS-Point 2.0, Anton Paar, Graz, Austria). The measurements were carried out in transmission mode. The SAXS camera was equipped with a micro-X-ray source operating at 50 W (point-focus). A two-dimensional (2D) X-ray detector (EIGER2 R 500K, Dectris, Switzerland) and Cu-Kα-radiation (*λ* = 0.1542 nm) were used. The sample-to-detector distance was 571 mm. All isotropic 2D patterns were azimuthally averaged to 1D SAXS curves, and the SAXS curves of pure solvent were subtracted. The angular *q*-range was 0.01 nm^−1^ to 6 nm^−1^, with *q* being the magnitude of the scattering vector, corresponding to a total 2*θ* region of 0.14–7. The exposure time was 3 × 300 s. A quartz capillary (1 mm diameter, 10 μm wall thickness) with two vacuum-tight screw caps on both ends was used as the sample cell and inserted into a thermostatted sample stage set (30°C). The vacuum in the camera was ≈1 mbar. The obtained spectra were analyzed using Genfit software (71), in the *q*-range of 0.5 nm^−1^ to 2.5 nm^−1^. The chosen model describes electron density as a linear combination of three functions:

$$\rho \left(r\right)=\rho_{0}+\frac{1}{2}\sum_{i=1}^{3}\left(\rho_{i-1}-\rho_{i}\right)\left[\mathrm{erf}\left(\frac{r-R_{i}}{2^{\frac{1}{2}}\sigma_{i}}\right)-\mathrm{erf}\left(\frac{r+R_{i}}{2^{\frac{1}{2}}\sigma_{i}}\right)\right]\text{,}$$

where *R*_*i*_ and *σ*_*i*_ correspond to positions and standard deviations of *i*-th step of the error function. In this model, parameters *ρ*_*i*_, *R*_*i*_, and *σ*_i_ are fitted. In the case of multilamellar liposomes, intensity depends on the structure factor, which was described using modified Caillé theory (72).

### Electron paramagnetic resonance

The concentration of liposomes for EPR measurements was 4 mg mL^−1^. EPR measurements were carried out on benchtop Bruker Magnettech ESR5000 spectrometer (Bruekr BioSpin, Germany). Measurement parameters were as follows: microwave frequency was 100 kHz, magnetic field modulation amplitude was 0.1 mT, and microwave power was 10 mW. EPR spectral simulations due to dynamics, and slow and fast motion, were carried out using Easyspin software (73) working at MATLAB platform (74).

### Atomic force microscopy

The method used to prepare supported lipid bilayers (SLBs) was the drop deposition method. Briefly, a suspension of multilamellar vesicles (100 μl) was added to the fluid cell containing a freshly cleaved mica plate. The system was left for 10 min to achieve adsorption. Owing to electrostatic interactions between liposomes and mica, solid lipid bilayers were formed. The nonadsorbed liposomes were removed by washing the surface with filtered PBS solution. The system was thermostatted at 25°C. The final concentration of lipid used for AFM measurements was 1 mg mL^−1^. The Bruker Dimension FastScan Bio AFM microscope (Bruker Nano Surfaces, Santa Barbara, CA) was used to study the structure and mechanical properties of the bilayer. The ScanAsyst Fluid+ silicon nitride cantilever (Bruker Nano Surfaces), equipped with a super sharp tip (nominal tip radius 2 nm), was used in a peak force quantitative nanomechanics mode. The deflection sensitivity and spring constant of the cantilever were calibrated using the in-built calibration manager of the Bruker NanoScope software. First, the initial values of the parameters were calculated from the thermal noise spectra. The exact calibration values were determined from the 30 nm long force-displacement curves when the set point 0.2 V was used. The typical deflection sensitivity was in the range of 34.1–39.3 nm V^−1^, and the spring constant of the cantilever was mostly found in the range of 0.71–0.92 N m^−1^. The following parameters were used when the peak force quantitative nanomechanics mode was used for combined surface characterization: peak force set point 200 pN, feedback gain was automatically set by the ScanAsyst algorithm of the NanoScope software. The scan speed was 1 Hz, the peak force frequency was 2 kHz, the peak force amplitude was 20 nm, and the Z-piezo scale was 0.5 um; (13 × 13) μm^2^ area view images, followed by (4×4) μm^2^ details, both with a resolution of 512 × 512 pixels. Thickness, roughness, and Young's modulus of the lipid bilayer were calculated using built-in software tools (75, 76, 77). Briefly, the thickness was determined from the force curves obtained on SLB. In the approaching force curve, the breakthrough feature (known as a jump) corresponds to the penetration of the bilayer (yield threshold force) and the width corresponds to the thickness of the bilayer. The roughness of the different domains of the SLBs has been obtained by four random average root mean square (*R*_a_) values on area of analyzed SLB. All images were presented as raw data except for the first-order 2D flattening. Force microscopy was used to study the elasticity of the SLB. From the resulting elasticity maps, Young's modulus values were determined. All results are presented as mean values with corresponding standard errors.

## Results

### Molecular dynamics simulations

**Fig. 2:**
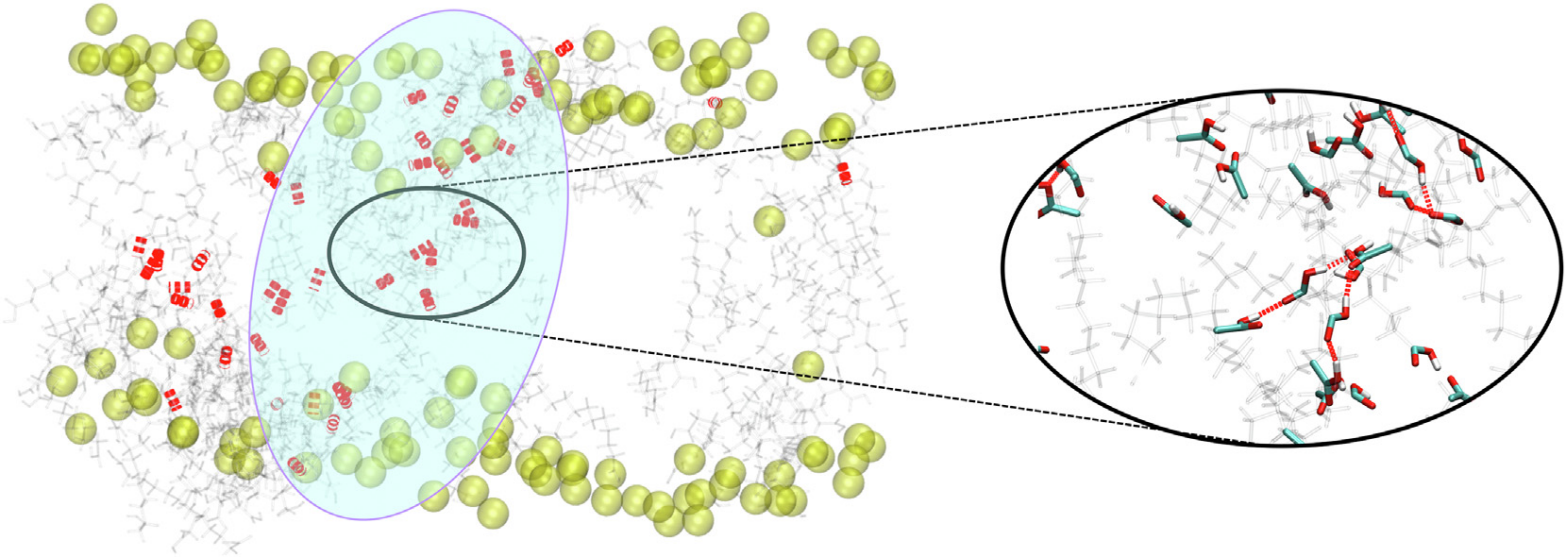
Snapshot of the DOPC-DDA system, with the focus on showing hydrogen bonds formed between carboxyl groups of DDA molecules (intermolecular DDA-DDA hydrogen bonds), appearing predominantly in DDA aggregate (average number of hydrogen bonds between all DDA molecules in DOPC-DDA system is 20.6 ± 4.1 during the last 150 ns, with them appearing predominantly in DDA aggregates). Hydrogen bonds are shown in red bold dashed lines. Blue ellipse highlights DDA-rich region/aggregate, virtually protruding throughout the bilayer. Phosphorous atoms are shown in spherical representation and colored in transparent yellow, with DDA being given in light gray. Inset shows close-up view of DDA molecules forming hydrogen bond bridges in the central region of the largest DDA aggregate.

**Fig. 3:**
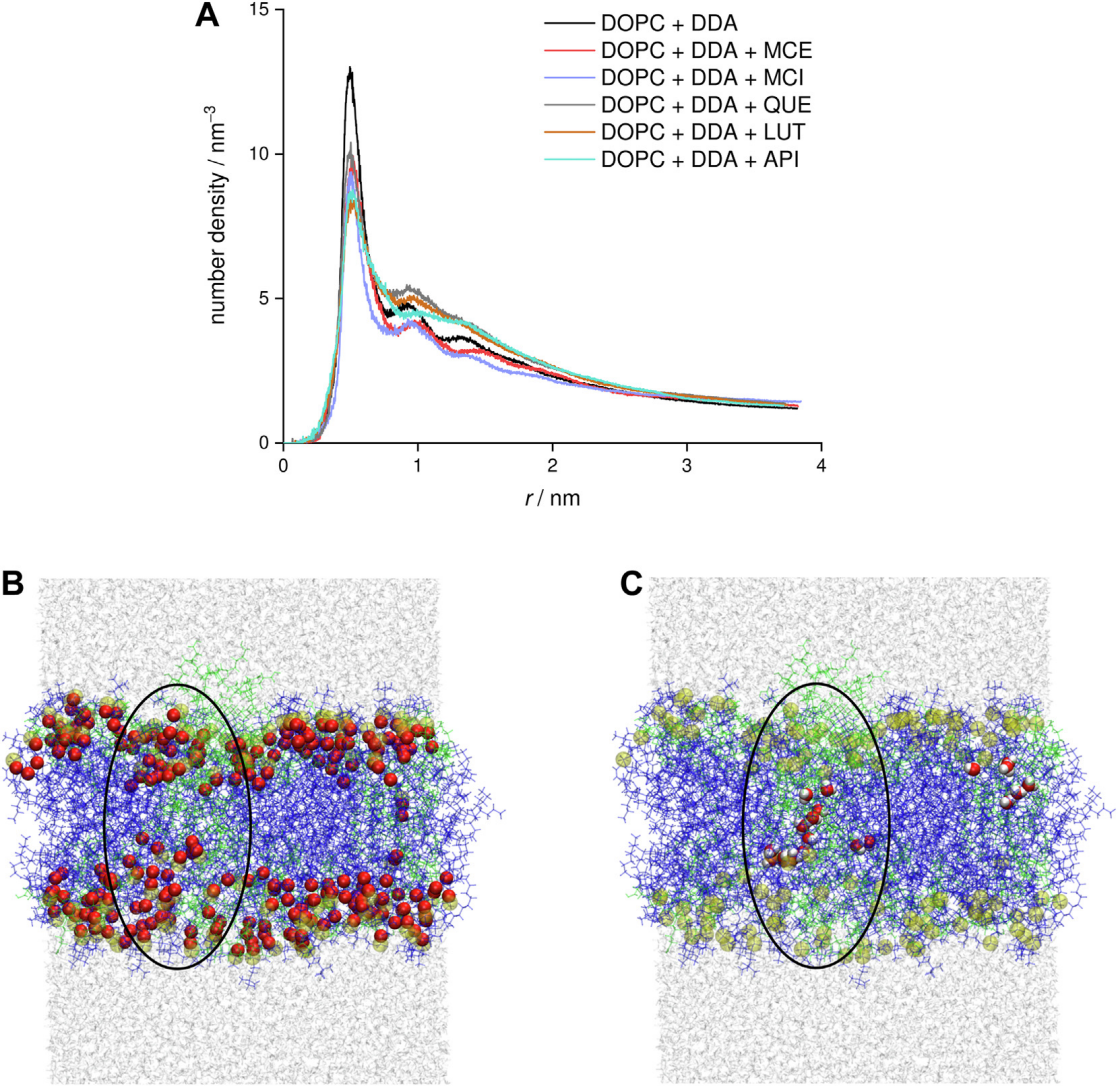
A: Radial distribution functions of DDA in DOPC systems with and without added flavonoids, based on the centers of mass of DDA molecules. B: Snapshot of DOPC-DDA system with oxygen atoms of DDA given in red spherical representation. C: Snapshot of the DOPC-DDA system with water molecules in the bilayer drawn in spherical representation. Water wire (indicated with black ellipse) forms during the first 100 ns of the production simulation and represents the onset of water pore formation. DOPC lipid is given in blue, DDA is shown in green, phosphorous atoms are shown in transparent yellow, while the remainder of water molecules is presented in light gray.

We first investigated the behavior of bilayers when only the model product of oxidative stress, i.e., DDA, is present. We observed that part of the DDA molecules is situated in the polar region of the lipid bilayer. This behavior is similar to the one observed in the case of POLMs (27, 32) and is a consequence of favorable interactions between carboxyl and phosphate groups. Correspondingly to the case of POLMs, the insertion of DDA reduces the interactions between the polar heads, as the present carboxyl groups also tend to interdigitate between lipid heads of DOPC (supplemental Fig. S1). However, in POLMs, hydrophobic chains rotate to move oxidized moieties near the polar head groups, resulting in interdigitation of lipid chains, whereas there is no interdigitation in the studied system. This is due to the fact that DDA is not covalently bonded to the phosphate groups and can position itself with greater conformational freedom in the bilayer. In addition, we find that DDA is partially inserted into the hydrophobic region of the bilayer, where it tends to form large aggregates (Fig. 2), which is easily observed from the large values of the calculated radial distribution function at small distances (Fig. 3A). Similarly, the DDA aggregate is clearly visible in Fig. 3B. From this snapshot, it is evident that the aggregate extends across the entire width of the membrane. Moreover, it can be seen that the carboxyl groups are partially located in the center of the bilayer, forming hydrogen-bonded clusters of DDA (Fig. 2).

**Fig. 4:**
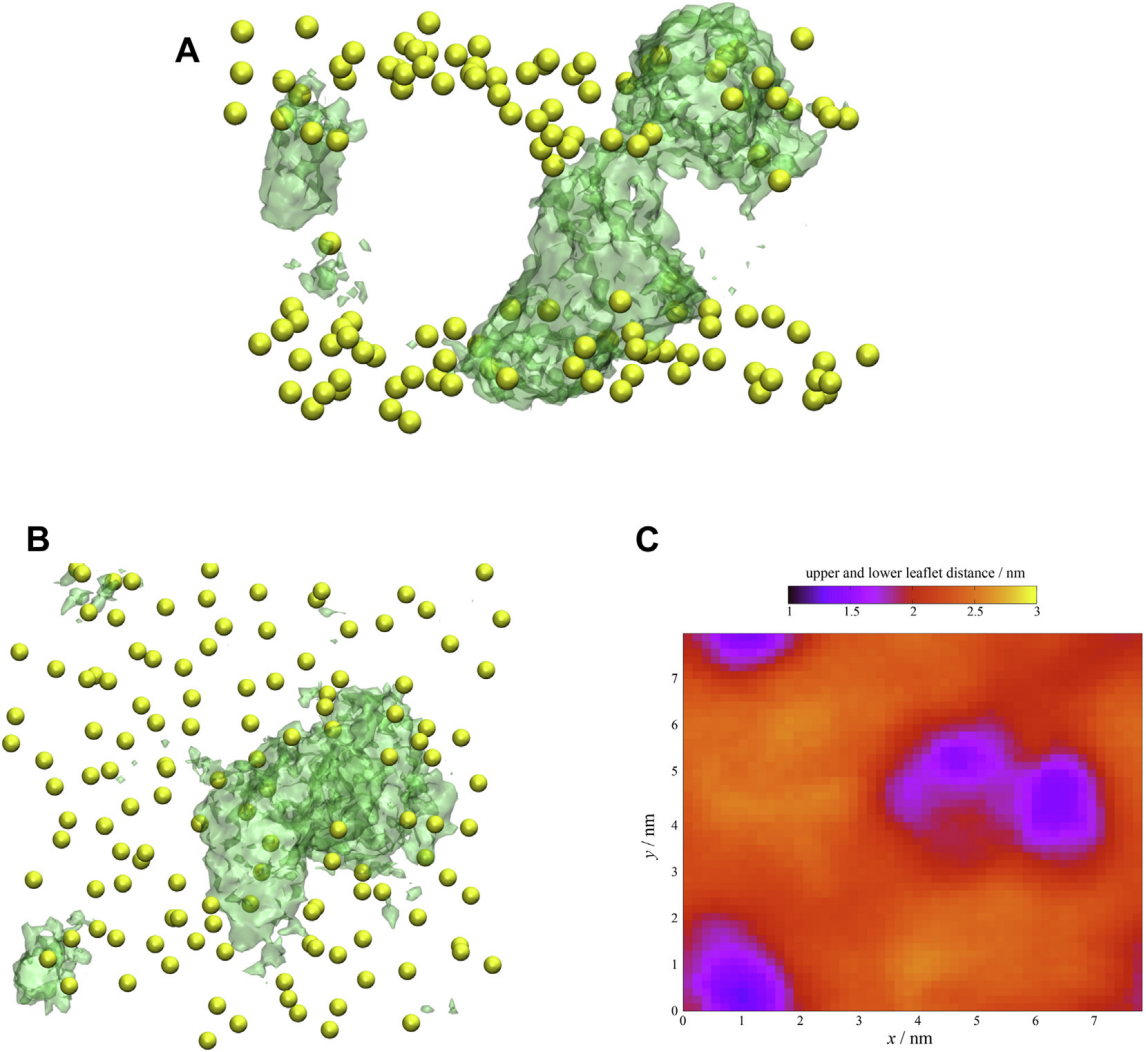
Average density map of DDA in the direction A: parallel and B: perpendicular to the bilayer for the DOPC-DDA system. Phosphorus atoms depicted as yellow transparent spheres, with DDA volumetric map given in green transparent representation (isovalue = 0.4). C: 2D heat map of average distance in the *z*-position between centers of mass of lipids belonging to the upper and the lower leaflet of the DOPC bilayer from the DOPC-DDA system, being in the same orientation as (B). Average density map and 2D heat map are calculated taking the last 50 ns of the simulation into consideration. Taking (B and C) together, one can directly infer that the lipid bilayer is significantly thinner exactly in the region where the DDA aggregate is formed (approximately 1.5 nm distance between the centers of mass of the upper and lower leaflet in the DDA aggregate region vs. ≈ 2.5 nm in the remainder of the bilayer).

**Fig. 5:**
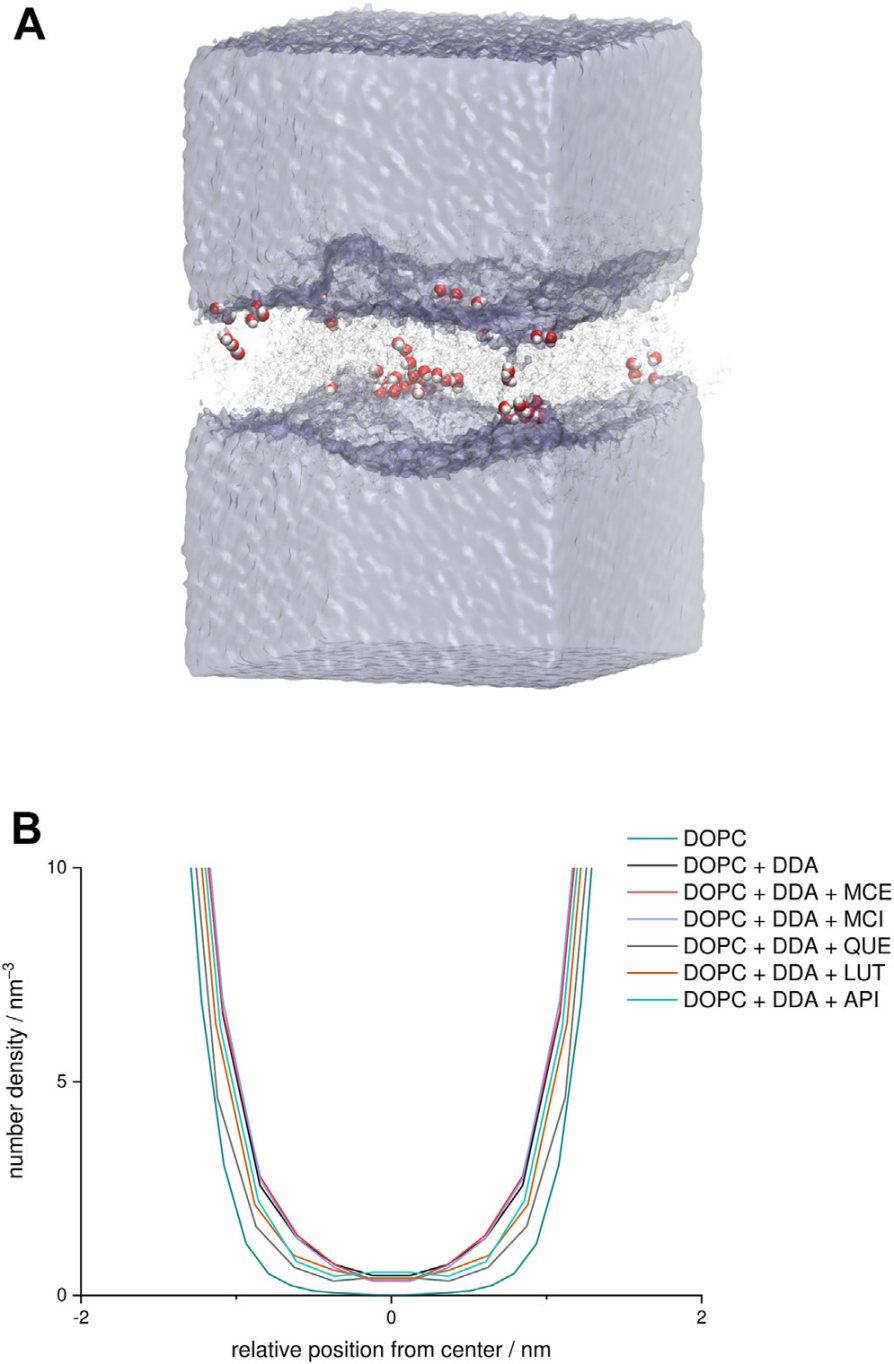
A: Snapshot of the DOPC-DDA system with average density maps of water (obtained considering the last 150 ns of their production runs). Membrane constituents are represented schematically, with a water volumetric map given in light blue transparent representation (isovalue = 0.2). Water molecules found inside the bilayer are shown in the sphere representation. B: Water distribution in systems with added DDA and flavonoids.

Since the formed aggregates have oxygen atoms in the middle of the bilayer, their polarity is increased, which may allow remarkable penetration of water deep into the membrane (Fig. 3C). On the other hand, in the DOPC bilayer without the DDA water does not penetrate beyond the polar groups (supplemental Fig. S2). Large aggregates formed by DDA have a profound effect on the bilayer, leading to large local deformations of the membrane (Figs. 4 and 5). This was additionally confirmed by a closer examination of the bilayer region in the vicinity of DDA. DOPC lipids located near the DDA aggregates show a different behavior compared with the region without aggregates. More precisely, lipid bilayer is significantly thinner in the regions dominated by DDA, with the distance between the upper and the lower leaflet of the bilayer being virtually 1 nm smaller compared with the remainder of the bilayer (Fig. 4C), having an impact on both the physical and chemical properties of the membrane. Previous research (31, 33, 34, 35) has shown that the incorporation of oxidized phosphocholine lipids into the membrane leads to the appearance of water clusters in the head group region and the formation of pores. These effects lead to increased permeability of the membrane (30), which may reduce its ability to act as a functional barrier. In our case, both the absence of oxo-group and relatively low concentrations of DDA led to subtler effects.

Although our simulations do not show the formation of pores extending from one side of the bilayer to the other, the observed aggregates and the resulting defects in the membrane structure mark the beginning of the pore formation process. This is an additional step in the mechanism of pore formation compared with POLMs bilayers.

**Fig. 6:**
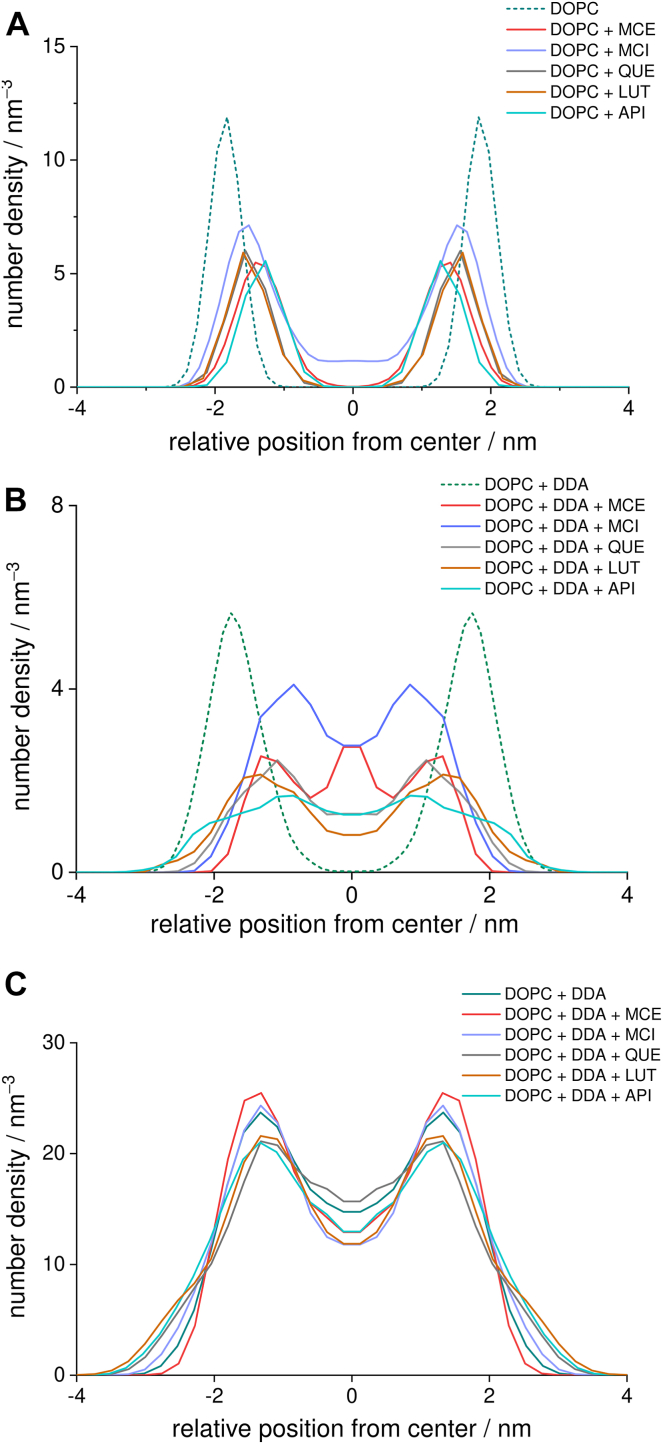
Number density profiles along the direction perpendicular to the bilayer for flavonoids in the DOPC system; flavonoid distribution (A) without and (B) with added DDA. Phosphate distribution in DOPC systems without flavonoids is shown as a reference (dashed). C: DDA distribution in systems with and without added flavonoids.

We now turn to the behavior of the membrane in the presence of selected flavonoids. From the number density profiles in Fig. 6A, it is evident that all flavonoids are mainly located near the polar groups of the lipids. This position correlates very well with the behavior of flavonoids in 1,2-dimyristoyl-sn-glycero-3-phosphocholine (DMPC) bilayers, where it was found that the sugar moiety in MCI forms more favorable hydrogen bonds with the surrounding water molecules and lipid heads (51). It is noteworthy that, in the case of MCI, there is a significant probability density in the center of the bilayer. This can be explained by its tendency to form small clusters, which is also evident from the radial distribution function (supplemental Fig. S3).

Formation of these clusters is driven by interactions between polar phenolic and hydroxyl groups in sugar moiety of different MCI molecules, which are more favorable than interactions between MCI and hydrophobic lipid chains. In addition, these clusters are expected to be less polar, facilitating their embedding into the hydrophobic region. In the systems containing flavonoids and DDA, an increase in the distribution of studied flavonoids is observed in the center of the bilayer (Fig. 6B), with a significant portion of flavonoids now occupying the central part of the membrane. It is interesting to note that a significant quantity of less polar flavonoids, QUE, API, and LUT, is positioned in the outer region of polar groups. In addition, we find that the equilibrium distribution of DDA changes with the introduction of flavonoids. All studied flavonoids except QUE were shown to decrease the quantity of DDA in the center of the bilayer (Fig. 6C). Less polar QUE, API, and LUT, additionally, force a portion of DDA molecules to positions on the outer parts of polar group region. We presume that this behavior is a result of more favorable interactions of phenol groups with polar heads in comparison with interactions of DDA, which result in DDA molecules being displaced either toward the aqueous medium, where they can form hydrogen bonds with water molecules, or, in the case of QUE, toward the center of the bilayer, where they can form hydrogen bonds with other DDA molecules. In addition, the results obtained indicate that there are two effects that determine the impact of flavonoids on the positioning of DDA in the hydrophobic region. Less polar flavonoids can penetrate the hydrophobic region more easily and interact with polar groups of DDA, displacing them from the center. Increasing the number of hydroxyl groups and polarity seems to decrease this impact at first, as evidenced by the case of QUE, while further increase of polarity, in case of MCE and MCI, drives the formation of less polar flavonoid aggregates that can reach the hydrophobic center of the membrane and displace the DDA molecules. The fact that all flavonoids perturb the DDA aggregates can be seen from the changes in the radial distribution function as well (Fig. 3A). Even more remarkable is the effect of DDA on the clustering of flavonoids. From the respective radial distribution functions and snapshots of the dynamics, it follows that, in the system with DDA, all flavonoids considered tend to form aggregates that cluster around the DDA-rich parts of the lipid membrane (supplemental Fig. S4) and compete with other components of the membrane (polar heads and water molecules) for interactions with the polar groups of DDA.

### Small-angle X-ray scattering

**Fig. 7:**
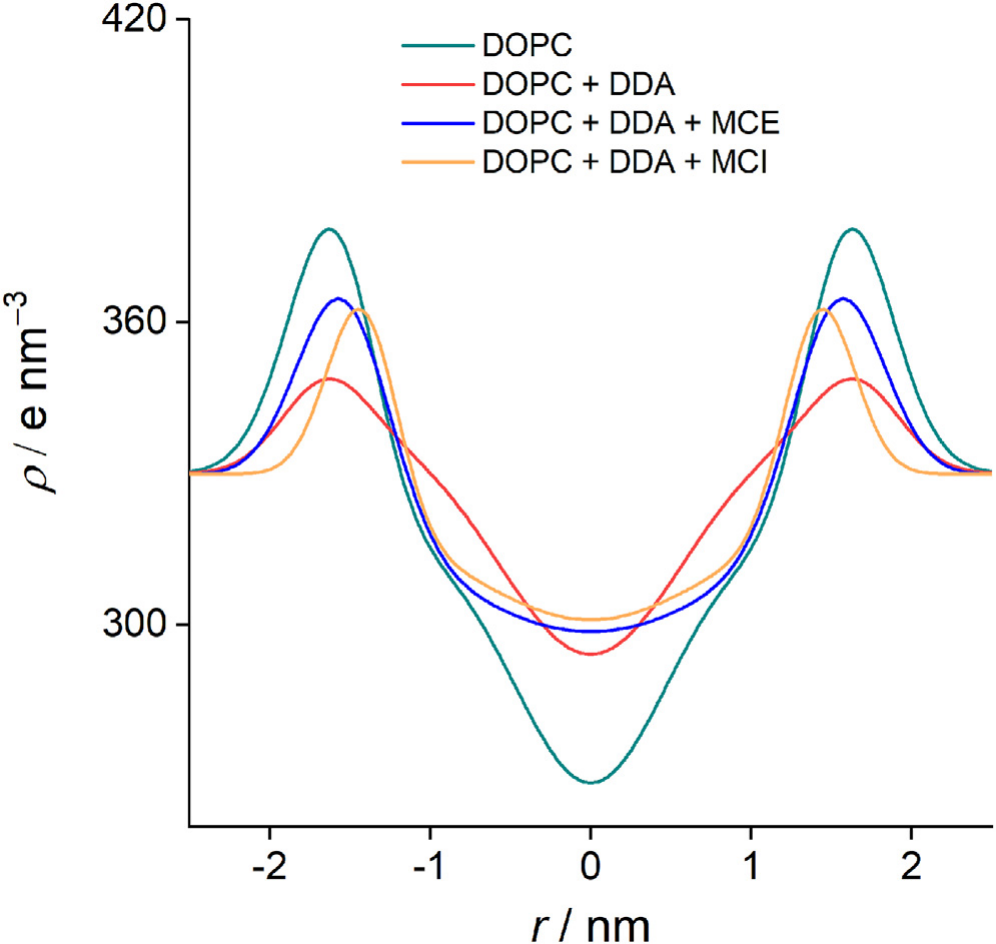
Electron density profiles of DOPC liposomes with and without DDA and flavonoids.

To further investigate the effect of DDA on the structural properties of bilayers, SAXS measurements were performed. The electron density profiles (Fig. 7) calculated from the SAXS curves exhibit the shape characteristic of phospholipid bilayers, with two peaks of electron density corresponding to phosphate head groups and a minimum in the center of the membrane, associated with the terminal methyl segments of phospholipid acyl chains (29).

The addition of DDA resulted in an increase in electron density in the middle of the bilayer, indicating the incorporation of alkyl chains and carboxyl groups of DDA into the central part of the membrane. This is consistent with the results of MD simulations, confirming that DDA chains extend through the nonpolar part of the membrane. Furthermore, electron density distribution corresponding to the polar head region becomes broader upon the addition of DDA. Moreover, electron density in this region decreases, which may be a consequence of incorporation of DDA molecules between the phosphate groups. Both effects are in accordance with the results obtained using MD simulations, specifically, the broadening of phosphate distribution and the position of the peaks of DDA distribution (Fig. 6).

The incorporation of flavonoids into the DDA-containing lipid membrane leads to an additional increase in electronic density in the hydrophobic region of the membrane (Fig. 7), indicating their incorporation in the center of the bilayer. Reduction of the electron density around 0.5 nm from the bilayer center indicates displacement of DDA molecules from the hydrophobic part of the membrane. In addition, the effect of DDA on the electron density in the polar head region is reduced in the systems containing MCE and MCI, indicating smaller effect of the interactions between polar heads and DDA.

### Electron paramagnetic resonance

The fluidity of lipid bilayers was studied by EPR, which is possible because the EPR spectrum of a magnetically anisotropic paramagnetic probe depends on the freedom of movement of the fatty acids (78). Because the internal dynamics of the bilayer correlates with the chemical composition and structure of the phospholipids, changes in the physical and chemical properties of the bilayer caused by oxidation can be detected by observing changes in the spectra of a paramagnetic probe. Quantitative analysis of 5-DSA-labeled samples has been performed by determining the order parameter, *S*, a measure of the local orientation order of the labeled molecule with respect to the normal to the surface of the bilayer. The value of the order parameter, *S*, close to 1.0 is characteristic of a more rigid lipid environment (79), whereas lower values of *S* are associated with fluid-like lipid phases (79, 80, 81, 82). As expected, the addition of DDA leads to a decrease in the order parameter, indicating an increase in membrane fluidity (Table 1). The slight decrease in *S* may be due to partial degradation of the membrane caused by the presence of the residual fatty acid molecules, i.e., DDA, which can rearrange the expected uniform distribution of 5-DSA among the available binding sites and result in loss of a small fraction of the fatty acid binding sites. Addition of flavonoids increases lipid order and reverses the aforementioned effect of DDA.

**Table 1:** Parameters derived from EPR spectra for 5-DSA-labeled samples

Sample	2*A*_||_/G	2*A*_⊥_/G	*S*	*τ*_c_^[a]^/ns
DOPC +5-DSA (control)	50.116	18.576	0.606	2.89
DOPC + 5-DSA + DDA	49.070	18.977	0.578	1.12
DOPC + 5-DSA + DDA + MCE	50.892	18.414	0.624	2.15
DOPC + 5-DSA + DDA + MCI	50.739	18.549	0.618	2.07
DOPC + 5-DSA + DDA + QUE	50.758	18.416	0.621	2.25
DOPC + 5-DSA + DDA + LUT	51.019	18.267	0.629	3.28
DOPC + 5-DSA + DDA + API	51.158	18.411	0.629	2.23

[a] Anisotropic rotation correlation time, τ_c_, given as the geometrically averaged value of diffusion tensor elements *D*_r_ = [*D*_x_*D*_y_*D*_z_]: $\tau_{c}=\frac{1}{6}∛D_{x}D_{y}D_{z}$ obtained by simulation of the EPR spectra.

The error of determined hyperfine constant, *A*, is related to the magnetic field resolution *B*(*x*_i_) = *B*_max_/*N*_max_ = 2.93 μT = 0.0293 G. *B*_max_ = 12 mT and *N*_max_ = 4,096, so Δ*A* = 0.029 G

### Atomic force microscopy

AFM was used to study morphological and nanomechanical properties of lipid bilayers after the addition of DDA and flavonoids.

**Fig. 8:**
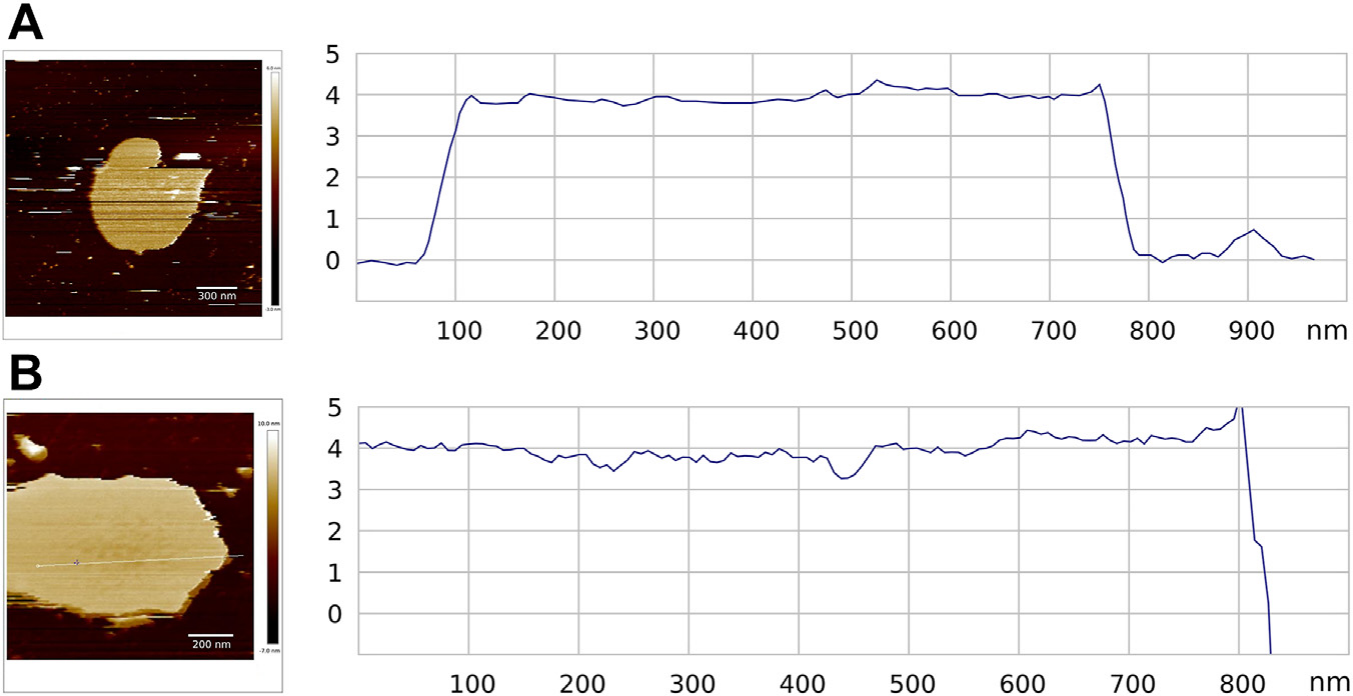
Cross sections of bilayers in (A) absence and (B) presence of DDA.

**Fig. 9:**
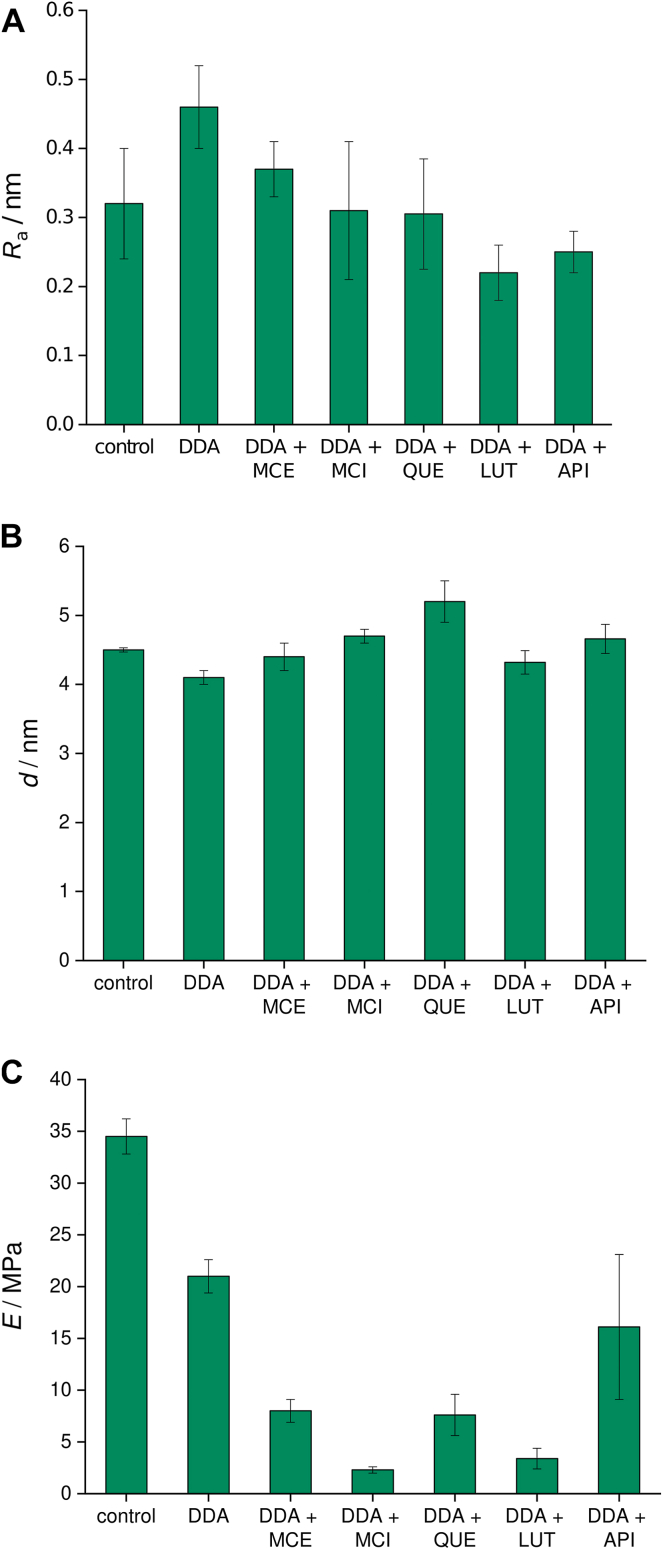
(A) Roughness, (B) thickness, and (C) elasticity of DOPC bilayers before and after the addition of DDA and flavonoids.

From the cross sections (Fig. 8), it is evident that the surface of the DOPC bilayer is relatively homogeneous, while the addition of DDA causes local perturbations and the appearance of defects. The calculation of the structural parameters (Fig. 9) shows that the most noticeable change caused by the addition of DDA is a decrease in elasticity (Fig. 9C), which is in accordance with previously reported results (83). This decrease confirms aggregation in the membranes, which has been shown to manifest itself in lower Young’s modulus values (84). Because added flavonoids also form hydrogen bonds and enable further aggregation, a decrease in Young’s moduli after the addition of flavonoids is expected. In addition, the most polar MCI has the greatest capacity to form hydrogen bonds, leading to a more prominent decrease in Young’s modulus.

The results for MCE and MCI are consistent with the presumed different interaction of aglycone and glycoside forms of the same flavonoid with lipid membranes (85). Strong interactions of the carboxyl groups of DDA with polar parts of lipid molecule have already been observed using other techniques and confirmed using AFM. An increase in the roughness is noted (Fig. 9A), suggesting that these interactions happen close to the surface of the bilayer. The increase caused by DDA is less pronounced in the presence of flavonoids, indicating their protective role and ability to maintain membrane integrity. The ability of lipid peroxidation products to form hydrogen bonds with a wide range of compounds (28), including water, carbonyl groups, and phosphate groups, was confirmed. Analogous to the changes in roughness, the thinning of the membrane caused by DDA is also less pronounced when flavonoids are added (Fig. 9B).

## Discussion

In this paper, we used DDA to simulate the effect of medium- to long-chain dicarboxylic acids, one of the products of oxidative stress, on lipid membrane properties. The effect of flavonoids on membrane functionality and integrity was also investigated.

Molecular simulations revealed specific interactions of DDA with the lipid bilayer. The carboxyl groups of DDA interact with the phosphate groups of the lipid molecule mainly via hydrogen bonds. As a result, part of the DDA molecules is located in the polar region of the bilayer. However, part of the DDA molecules was found to incorporate in the nonpolar region as well, with the carboxyl groups forming hydrogen-bonded clusters in the center of the bilayer. The incorporation of DDA in the nonpolar region was confirmed after observing electron density profiles. A similar increase in electron density in the center of the bilayer was observed upon oxidation of PLPC (30) and DLPC (29) lipids. However, in these cases, an increase was attributed to the interdigitation of lipid chains, which is not present in our system. In a similar system of POPC liposomes with POLMs terminated with carboxyl groups, De Rosa *et al.* (86) found that the distribution of carboxyl groups is bimodal. Part of the groups were located in the hydrophobic region of the membrane, while parts of the oxidized chains rotated to move the corresponding carboxyl group near the polar head. However, due to the limited movement of the oxidized chains caused by the covalent bond to the polar head, carboxyl groups in this system are not able to penetrate the center of the membrane. Since DDA has polar carboxyl groups at each end of the hydrocarbon chain, the interaction with polar parts of the membrane occurs on both sides of the bilayer. At the same time, the second carboxyl groups form hydrogen-bonded dimers in the lipophilic part of the membrane, leading to an increase in electron density in the center of the membrane. Such an increase is analogous to that observed in lipid membranes with incorporated transmembrane proteins, reported by Narayanan *et al.* (87). In addition, the electron density profile of DOPC with DDA shows broadening of the electron density in the region of polar groups toward the center of the bilayer. Such effect is in accordance with the MD results (Fig. 6) and is a combination of the electron density of incorporated DDA molecules and burying of the phosphate groups induced by its presence.

The clusters cause the appearance of defects in the bilayer. The occurrence of such defects was verified by an increase in surface roughness and a decrease in elasticity. In addition, a loss of order in the region of the polar heads was observed, which was previously associated with pore formation. The aggregates cause an increase in the polarity of the central part of the membrane, allowing water to penetrate deep into the membrane. Although we studied the subtle effects of DDA and did not observe complete pore formation, the penetration, together with the observed structural defects, is the beginning of the process. This step in the mechanism of pore formation is absent when using POLMs (30, 32), indicating significantly different behavior of peroxidation products of different structure.

The addition of flavonoids changes the equilibrium distribution of DDA, with a considerable portion of flavonoids occupying the central part of the membrane. This is reflected in the increase in electron density at the center of the membrane. This is in accordance with previously observed positioning of flavonoid molecules deeper in the barrier in the presence of DDA. Moreover, the addition of flavonoids appears to restore electron density profile near the region of polar groups, which is a consequence of either displacing DDA molecules toward the polar groups (as noticed from the MD distributions) or restoration of phosphate distribution. These results indicate competition for hydrogen bonding with DDA between flavonoids and polar heads. Diffusion of flavonoids deeper into the membrane is caused by their clustering, resulting in the formation of less polar aggregates compared with isolated molecules. In addition, all flavonoids tend to aggregate around the DDA-rich parts of the membrane. This behavior suggests that different components of the bilayer compete for interactions with polar groups of DDA.

EPR findings, namely, the dissipation of the anisotropic rotation correlation time, *τ*_c_ (Table 1), confirm that the molecule tumbles asymmetrically after the addition of DDA. In addition, a decrease in order parameter *S* could be a consequence of a disordered lipid phase in the vicinity of DDA aggregates, where MD simulations revealed that the phosphate groups are buried in the bilayer, implying that the lipid molecules are arranged in different orientations. These results are in accordance with the loss of anisotropy in EPR spectra of 5-DSA probe observed by Megli and Russo (26), who studied different model peroxidation products integrated into the membrane. Similar results were also reported by Tai *et al.* (88) and Beranova *et al.* (89), who studied oxidized phospholipid membranes. The latter study showed that even a small addition of oxidized phospholipid leads to increased mobility of head groups and lateral diffusion. Further confirmation using MD simulations was proposed by Van der Paal *et al.* (14), who found a decrease in order parameter in oxidized POPC liposomes. In the systems containing flavonoids and DDA, there is no decrease in the order parameter, while the changes upon addition of flavonoids are a consequence of the interactions with DDA and lipid head groups. These results confirm the competition of flavonoids and phosphate groups for hydrogen bonding with DDA. The formation of hydrogen bonds between flavonoids and polar head groups is of great importance because it can regulate the ability of flavonoids to interact with the membrane and protect its integrity (50). Finally, the interactions between flavonoids and DDA molecules reduce the changes in structural properties caused by DDA, ie, increase in surface roughness and decrease in the order parameter, indicating protective action on the membrane integrity.

It can be concluded that, while the main benefit of flavonoids is their great potential to counteract the deleterious effects of the uncontrolled generation of reactive oxygen species in the membrane through their ability to scavenge radicals, their physical properties could also potentially have a beneficial effect on membrane functionality, as they are able to repair the perturbations imposed on the membrane by the products of oxidative stress.

## Data Statement

The data supporting the findings of this study are available from the corresponding authors upon reasonable request.

## Supplemental data

This article contains supplemental data.

## Conflict of interest

The authors declare that they have no conflicts of interest with the contents of this article.

## References

[1] Sabatini K., Mattila J.P., Megli F.M., Kinnunen P.K.J. (2006). Characterization of two oxidatively modified phospholipids in mixed monolayers with DPPC. Biophys. J..

[2] Chabanon M., Stachowiak J.C., Rangamani P. (2017). Systems biology of cellular membranes: a convergence with biophysics. Wiley Interdiscip. Rev. Syst. Biol. Med..

[3] Fruhwirth G.O., Loidl A., Hermetter A. (2007). Oxidized phospholipids: from molecular properties to disease. Biochim. Biophys. Acta Mol. Basis Dis..

[4] McIntyre T.M., Zimmerman G.A., Prescott S.M. (1999). Biologically active oxidized phospholipids. J. Biol. Chem..

[5] Schnitzer E., Pinchuk I., Lichtenberg D. (2007). Peroxidation of liposomal lipids. Eur. Biophys. J..

[6] Parola M., Robino G. (2001). Oxidative stress-related molecules and liver fibrosis. J. Hepatol..

[7] Repetto M., Semprine J., Boveris A., Catala A. (2012). Lipid Peroxidation.

[8] Girotti A.W. (1998). Lipid hydroperoxide generation, turnover, and effector action in biological systems. J. Lipid Res..

[9] Stemmer U., Dunai Z.A., Koller D., Pürstinger G., Zenzmaier E., Deigner H.P. (2012). Toxicity of oxidized phospholipids in cultured macrophages. Lipids Health Dis..

[10] Mitchell A.E., Morin D., Lame M.W., Jones A.D. (1995). Purification, mass spectrometric characterization, and covalent modification of murine glutathione S-transferases. Chem. Res. Toxicol..

[11] Vander Jagt D.L., Hunsaker L.A., Vander Jagt T.J., Gomez M.S., Gonzales D.M., Deck L.M. (1997). Inactivation of glutathione reductase by 4-hydroxynonenal and other endogenous aldehydes. Biochem. Pharmacol..

[12] Pospíšil P., Yamamoto Y. (2017). Damage to photosystem II by lipid peroxidation products. Biochim. Biophys. Acta Gen. Subj..

[13] Song H., Amati A., Pannwitz A., Bonnet S., Hammarström L. (2022). Mechanistic insights into the charge transfer dynamics of photocatalytic water oxidation at the lipid bilayer–water interface. J. Am. Chem. Soc..

[14] Van Der Paal J., Neyts E.C., Verlackt C.C.W., Bogaerts A. (2016). Effect of lipid peroxidation on membrane permeability of cancer and normal cells subjected to oxidative stress. Chem. Sci..

[15] Caetano W., Haddad P.S., Itri R., Severino D., Vieira V.C., Baptista M.S. (2007). Photo-induced destruction of giant vesicles in methylene blue solutions. Langmuir.

[16] Haluska C.K., Baptista M.S., Fernandes A.U., Schroder A.P., Marques C.M., Itri R. (2012). Photo-activated phase separation in giant vesicles made from different lipid mixtures. Biochim. Biophys. Acta Biomembr..

[17] Volinsky R., Paananen R., Kinnunen P.K.J. (2012). Oxidized phosphatidylcholines promote phase separation of cholesterol-sphingomyelin domains. Biophys. J..

[18] Domínguez R.O., Marschoff E.R., Guareschi E.M., Repetto M.G., Famulari A.L., Pagano M.A. (2008). Insulin, glucose and glycated hemoglobin in Alzheimer’s and vascular dementia with and without superimposed Type II diabetes mellitus condition. J. Neural Transm..

[19] Famulari A.L., Marschoff E.R., Llesuy S.F., Kohan S., Serra J.A., Dominguez R.O. (1996). The antioxidant enzymatic blood profile in Alzheimer’s and vascular diseases. Their association and a possible assay to differentiate demented subjects and controls. J. Neurol. Sci..

[20] Blesa J., Trigo-Damas I., Quiroga-Varela A., Jackson-Lewis V.R. (2015). Oxidative stress and Parkinson’s disease. Front. Neuroanat..

[21] Repetto M., Maria A., Guzmán J., Giordano O., Llesuy S. (2010). Protective effect of Artemisia douglasiana Besser extracts in gastric mucosal injury. J. Pharm. Pharmacol..

[22] Repetto M.G., Ossani G., Monserrat A.J., Boveris A. (2010). Oxidative damage: the biochemical mechanism of cellular injury and necrosis in choline deficiency. Exp. Mol. Pathol..

[23] Reis A., Domingues P., Ferrer-Correia A.J.V., Domingues M.R.M. (2004). Fragmentation study of short-chain products derived from oxidation of diacylphosphatidylcholines by electrospray tandem mass spectrometry: identification of novel short-chain products. Rapid Commun. Mass Spectrom..

[24] Sadžak A., Mravljak J., Maltar-Strmečki N., Arsov Z., Baranović G., Erceg I. (2020). The structural integrity of the model lipid membrane during induced lipid peroxidation: the role of flavonols in the inhibition of lipid peroxidation. Antioxidants.

[25] Parra-Ortiz E., Browning K.L., Damgaard L.S.E., Nordström R., Micciulla S., Bucciarelli S. (2019). Effects of oxidation on the physicochemical properties of polyunsaturated lipid membranes. J. Colloid Interf. Sci..

[26] Megli F.M., Russo L. (2008). Different oxidized phospholipid molecules unequally affect bilayer packing. Biochim. Biophys. Acta Biomembr..

[27] Itri R., Junqueira H.C., Mertins O., Baptista M.S. (2014). Membrane changes under oxidative stress: the impact of oxidized lipids. Biophys. Rev..

[28] Siani P., de Souza R.M., Dias L.G., Itri R., Khandelia H. (2016). An overview of molecular dynamics simulations of oxidized lipid systems, with a comparison of ELBA and MARTINI force fields for coarse grained lipid simulations. Biochim. Biophys. Acta Biomembr..

[29] Mason R.P., Walter M.F., Mason P.E. (1997). Effect of oxidative stress on membrane structure: small-Angle X-Ray diffraction analysis. Free Radic. Biol. Med..

[30] Wong-ekkabut J., Xu Z., Triampo W., Tang I.-M., Peter Tieleman D., Monticelli L. (2007). Effect of lipid Peroxidation on the properties of lipid bilayers: a molecular dynamics study. Biophys. J..

[31] Runas K.A., Malmstadt N. (2015). Low levels of lipid oxidation radically increase the passive permeability of lipid bilayers. Soft Matter.

[32] Cwiklik L., Jungwirth P. (2010). Massive oxidation of phospholipid membranes leads to pore creation and bilayer disintegration. Chem. Phys. Lett..

[33] Conte E., Megli F.M., Khandelia H., Jeschke G., Bordignon E. (2013). Lipid peroxidation and water penetration in lipid bilayers: a W-band EPR study. Biochim. Biophys. Acta Biomembr..

[34] Lis M., Wizert A., Przybylo M., Langner M., Swiatek J., Jungwirth P. (2011). The effect of lipid oxidation on the water permeability of phospholipids bilayers. Phys. Chem. Chem. Phys..

[35] Vernier P.T., Levine Z.A., Wu Y.H., Joubert V., Ziegler M.J., Mir L.M. (2009). Electroporating fields target oxidatively damaged areas in the cell membrane. PLoS One.

[36] Reis A., Domingues M.R.M., Amado F.M.L., Ferrer-Correia A.J.V., Domingues P. (2005). Separation of peroxidation products of diacyl-phosphatidylcholines by reversed-phase liquid chromatography-mass spectrometry. Biomed. Chromatogr..

[37] Inouye M., Mio T., Sumino K. (2000). Dicarboxylic acids as markers of fatty acid peroxidation in diabetes. Atherosclerosis.

[38] Passi S., Picardo M., De Luca C., Nazzaro-Porro M., Rossi L., Rotilio G. (1993). Saturated dicarboxylic acids as products of unsaturated fatty acid oxidation. Biochim. Biophys. Acta Lipids Lipid Metab..

[39] Reis A., Spickett C.M. (2012). Chemistry of phospholipid oxidation. Biochim. Biophys. Acta Biomembr..

[40] Havsteen B.H. (2002). The biochemistry and medical significance of the flavonoids. Pharmacol. Ther..

[41] Valko M., Morris H., Cronin M. (2005). Metals, toxicity and oxidative stress. Curr. Med. Chem..

[42] El-Beltagi H.S., Mohamed H.I. (2013). Reactive oxygen species, lipid peroxidation and antioxidative defense mechanism. Not Bot. Horti Agrobot Cluj Napoca.

[43] Terao J., Piskula M., Yao Q. (1994). Protective effect of epicatechin, epicatechin gallate, and quercetin on lipid peroxidation in phospholipid bilayers. Arch. Biochem. Biophys..

[44] Afanas’ev I.B., Dcrozhko A.I., Brodskii A.V., Kostyuk V.A., Potapovitch A.I. (1989). Chelating and free radical scavenging mechanisms of inhibitory action of rutin and quercetin in lipid peroxidation. Biochem. Pharmacol..

[45] Panche A.N., Diwan A.D., Chandra S.R. (2016). Flavonoids: an overview. J. Nutr. Sci..

[46] Sinha R., Joshi A., Joshi U.J., Srivastava S., Govil G. (2014). Localization and interaction of hydroxyflavones with lipid bilayer model membranes: a study using DSC and multinuclear NMR. Eur. J. Med. Chem..

[47] Saija A., Scalese M., Lanza M., Marzullo D., Bonina F., Castelli F. (1995). Flavonoids as antioxidant agents: importance of their interaction with biomembranes. Free Radic. Biol. Med..

[48] Košinová P., Berka K., Wykes M., Otyepka M., Trouillas P. (2012). Positioning of antioxidant quercetin and its metabolites in lipid bilayer membranes: implication for their lipid-peroxidation inhibition. J. Phys. Chem. B.

[49] Ollila F., Halling K., Vuorela P., Vuorela H., Slotte J.P. (2002). Characterization of flavonoid-biomembrane interactions. Arch. Biochem. Biophys..

[50] Oteiza P.I., Erlejman A.G., Verstraeten S.V., Keen C.L., Fraga C.G. (2005). Flavonoid-membrane interactions: a protective role of flavonoids at the membrane surface?. Clin. Dev. Immunol..

[51] Sadžak A., Brkljača Z., Crnolatac I., Baranović G., Šegota S. (2020). Flavonol clustering in model lipid membranes: DSC, AFM, force spectroscopy and MD simulations study. Colloids Surf. B Biointer..

[52] Pohjala L., Tammela P. (2012). Aggregating behavior of phenolic compounds — a source of false bioassay results?. Molecules.

[53] Sirk T.W., Brown E.F., Friedman M., Sum A.K. (2009). Molecular binding of catechins to biomembranes: relationship to biological activity. J. Agric. Food Chem..

[54] Chung M.Y., Hwang J.T., Lee J., Choi H.K. (2022).

[55] Dabeek W.M., Marra M.V. (2019). Dietary quercetin and Kaempferol: bioavailability and potential cardiovascular-related bioactivity in humans. Nutrients.

[56] Formica J.V., Regelson W. (1995). Review of the biology of quercetin and related bioflavonoids. Food Chem. Toxicol..

[57] Liu S., Zhu Y., Liu N., Fan D., Wang M., Zhao Y. (2021). Antioxidative properties and chemical changes of quercetin in fish oil: quercetin reacts with free fatty acids to form its ester derivatives. J. Agric. Food Chem..

[58] Pham T.N.D., Stempel S., Shields M.A., Spaulding C., Kumar K., Bentrem D.J. (2019). Quercetin enhances the anti-tumor effects of BET inhibitors by suppressing hnRNPA1. Int. J. Mol. Sci..

[59] Sekher Pannala A., Chan T.S., O’Brien P.J., Rice-Evans C.A. (2001). Flavonoid B-Ring chemistry and antioxidant activity: fast reaction kinetics. Biochem. Biophys. Res. Commun..

[60] Lefort É.C., Blay J. (2013). Apigenin and its impact on gastrointestinal cancers. Mol. Nutr. Food Res..

[61] Majma Sanaye P., Mojaveri M.R., Ahmadian R., Sabet Jahromi M., Bahramsoltani R. (2022). Apigenin and its dermatological applications: a comprehensive review. Phytochemistry.

[62] Zhu L.-H., Bi W., Qi R.-B., Wang H.-D., Wang Z.-G., Zeng Q. (2011). Luteolin reduces primary hippocampal neurons death induced by neuroinflammation. Neurol. Res..

[63] Seelinger G., Merfort I., Wölfle U., Schempp C. (2008). Anti-carcinogenic effects of the flavonoid Luteolin. Molecules.

[64] Bangham A.D., De Gier J., Greville G.D. (1967). Osmotic properties and water permeability of phospholipid liquid crystals. Chem. Phys. Lipids.

[65] Fukumoto L.R., Mazza G. (2000). Assessing antioxidant and prooxidant activities of phenolic Compounds. J. Agric. Food Chem..

[66] Ermilova I., Lyubartsev A.P. (2016). Extension of the Slipids force field to polyunsaturated lipids. J. Phys. Chem. B.

[67] Jämbeck J.P.M., Lyubartsev A.P. (2012). Derivation and systematic validation of a refined all-atom force field for phosphatidylcholine lipids. J. Phys. Chem. B.

[68] Jämbeck J.P.M., Lyubartsev A.P. (2012). An extension and further validation of an all-atomistic force field for biological membranes. J. Chem. Theor. Comput..

[69] Jämbeck J.P.M., Lyubartsev A.P. (2013). Another piece of the membrane puzzle: extending Slipids further. J. Chem. Theor. Comput..

[70] Martínez L., Andrade R., Birgin E.G., Martínez J.M. (2009). PACKMOL: a package for building initial configurations for molecular dynamics simulations. J. Comput. Chem..

[71] Spinozzi F., Ferrero C., Ortore M.G., De Maria Antolinos A., Mariani P. (2014). GENFIT: software for the analysis of small-angle X-ray and neutron scattering data of macromolecules in solution. J. Appl. Crystallogr..

[72] Zhang R., Suter R.M., Nagle J.F. (1994). Theory of the structure factor of lipid bilayers. Phys. Rev. E.

[73] Stoll S., Schweiger A. (2006). EasySpin, a comprehensive software package for spectral simulation and analysis in EPR. J. Magn. Reson..

[74] (2011). MATLAB, R2011b.

[75] Bruker (2011). Nanoscope Analysis 1.50 User Manual.

[76] Jazvinšćak Jembrek M., Šimić G., Hof P.R., Šegota S. (2015). Atomic force microscopy as an advanced tool in neuroscience. Transl. Neurosci..

[77] Jazvinšćak Jembrek M., Vlainić J., Čadež V., Šegota S. (2018). Atomic force microscopy reveals new biophysical markers for monitoring subcellular changes in oxidative injury: neuroprotective effects of quercetin at the nanoscale. PLoS One.

[78] Jurkiewicz P., Olżyńska A., Cwiklik L., Conte E., Jungwirth P., Megli F.M. (2012). Biophysics of lipid bilayers containing oxidatively modified phospholipids: insights from fluorescence and EPR experiments and from MD simulations. Biochim. Biophys. Acta Biomembr..

[79] Gaffney B.J., Berliner L.J. (1976). Spin labeling: Theory and applications.

[80] Broido M.S., Meirovitch E. (1983). Doxyl nitroxide probes and the intrinsic flexibility gradient. A slow-motional line shape analysis study. J. Phys. Chem..

[81] Chede L.S., Wagner B.A., Buettner G.R., Donovan M.D. (2021). Electron spin resonance evaluation of buccal membrane fluidity alterations by sodium caprylate and L-menthol. Int. J. Mol. Sci..

[82] Hubbel W., McConnell H. (1971). Molecular motion in spin-labeled phospholipids and membranes. J. Am. Chem. Soc..

[83] Eid J., Jraij A., Greige-Gerges H., Monticelli L. (2021). Effect of quercetin on lipid membrane rigidity: assessment by atomic force microscopy and molecular dynamics simulations. BBA Adv..

[84] Ungureanu A.-A., Benilova I., Krylychkina O., Braeken D., De Strooper B., Van Haesendonck C. (2016). Amyloid beta oligomers induce neuronal elasticity changes in age-dependent manner: a force spectroscopy study on living hippocampal neurons. Sci. Rep..

[85] Londoño-Londoño J., De Lima V.R., Jaramillo C., Creczynski-pasa T. (2010). Hesperidin and hesperetin membrane interaction: understanding the role of 7-O-glycoside moiety in flavonoids. Arch. Biochem. Biophys..

[86] De Rosa R., Spinozzi F., Itri R. (2018). Hydroperoxide and carboxyl groups preferential location in oxidized biomembranes experimentally determined by small angle X-ray scattering: implications in membrane structure. Biochim. Biophys. Acta Biomembr..

[87] Narayanan T., Weerakkody D., Karabadzhak A.G., Anderson M., Andreev O.A., Reshetnyak Y.K. (2016). pHLIP peptide interaction with a membrane monitored by SAXS. J. Phys. Chem. B.

[88] Tai W.Y., Yang Y.C., Lin H.J., Huang C.P., Cheng Y.L., Chen M.F. (2010). Interplay between structure and fluidity of model lipid membranes under oxidative attack. J. Phys. Chem. B.

[89] Beranova L., Cwiklik L., Jurkiewicz P., Hof M., Jungwirth P. (2010). Oxidation changes physical properties of phospholipid bilayers: fluorescence spectroscopy and molecular simulations. Langmuir.

