# Supplementary material for: Puncturing lipid membranes: onset of pore formation and the role of hydrogen bonding in the presence of flavonoids
Source: J Lipid Res. 2023 Aug 22;64(10):100430. doi: 10.1016/j.jlr.2023.100430 (PMC10518586; doi:10.1016/j.jlr.2023.100430)
Supplement: Supplementary data [file mmc1.docx]

SUPPLEMENTARY MATERIAL

Puncturing lipid membranes: Onset of pore formation and the role of hydrogen bonding in the presence of flavonoids

Anja Sadžak*^a^, Zlatko Brkljača^b^, Mihael Eraković^a^, Manfred Kriechbaum^c^, Nadica Maltar-Strmečki^a^, Jan Přibyl^d^ and Suzana Šegota*^a^

a Division of Physical Chemistry, Ruđer Bošković Institute, Bijenička Cesta 54, 10000 Zagreb, Croatia
e-mail: [asadzak@irb.hr](mailto:asadzak@irb.hr), [merakov@irb.hr](mailto:merakov@irb.hr), [nstrm@irb.hr](mailto:nstrm@irb.hr), [ssegota@irb.hr](mailto:ssegota@irb.hr)

b Division of Organic Chemistry and Biochemistry, Ruđer Bošković Institute, Bijenička Cesta 54, 10000 Zagreb, Croatia
e-mail: [zlatko.brkljaca@selvita.com](mailto:zlatko.brkljaca@selvita.com)

c Institute of Inorganic Chemistry, Graz University of Technology, Stremayrgasse 9, 8010 Graz, Austria
e-mail: [manfred.kriechbaum@tugraz.at](mailto:manfred.kriechbaum@tugraz.at)

d CEITEC MU, Masaryk University, Kamenice 753/5 6, 2500 Brno, Czech Republic
e-mail: [jan.pribyl@ceitec.muni.cz](mailto:jan.pribyl@ceitec.muni.cz)


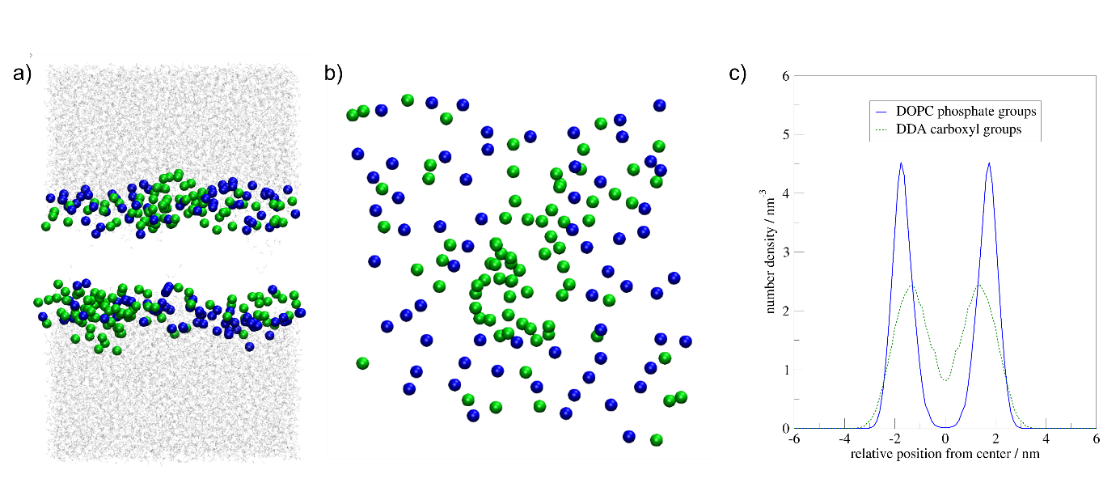


**Figure S1.** a) Snapshot of DOPC-DDA system. b) Upper leaflet of the bilayer viewed along the perpendicular direction to the bilayer, i.e., along the *z*-direction (view from above). Phosphorous atoms are shown in green, carbon atoms belonging to carboxyl groups of DDA are shown in blue, with water molecules given in the transparent gray representation. To showcase the interdigitation of carboxyl groups between polar lipid heads, only carboxyl groups belonging to the polar region of the bilayer are shown (buried carboxyl groups omitted for clarity). c) Number density profiles in the direction perpendicular to the bilayer, for phosphate and carboxyl groups stemming from DOPC and DDA, respectively. Only oxygen atoms belonging to these groups are considered in calculation of the distributions.


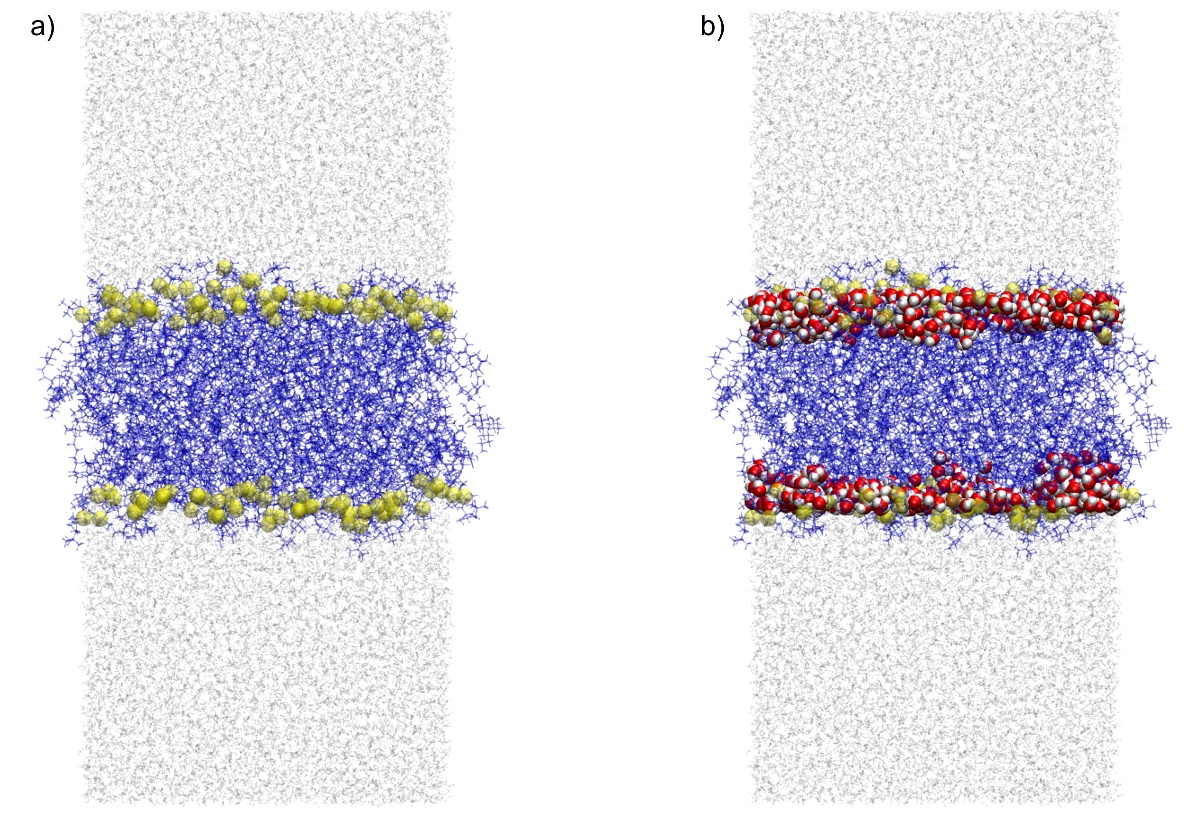


**Figure S2**. Snapshots of DOPC system with the highlight on a) polar lipid heads and b) water molecules buried in the lipid headgroup region. DOPC lipid is given in blue, phosphorous atoms are shown in transparent yellow, highlighted water molecules are shown in spherical representation, while the remainder of water molecules is presented in light grey. As can be observed from the presented snapshots, as well as from Figure 2b (see article), water molecules do not penetrate the hydrophobic region of the bilayer, i.e., neither water pore formation nor its onset can be observed in the simulation of DOPC system.


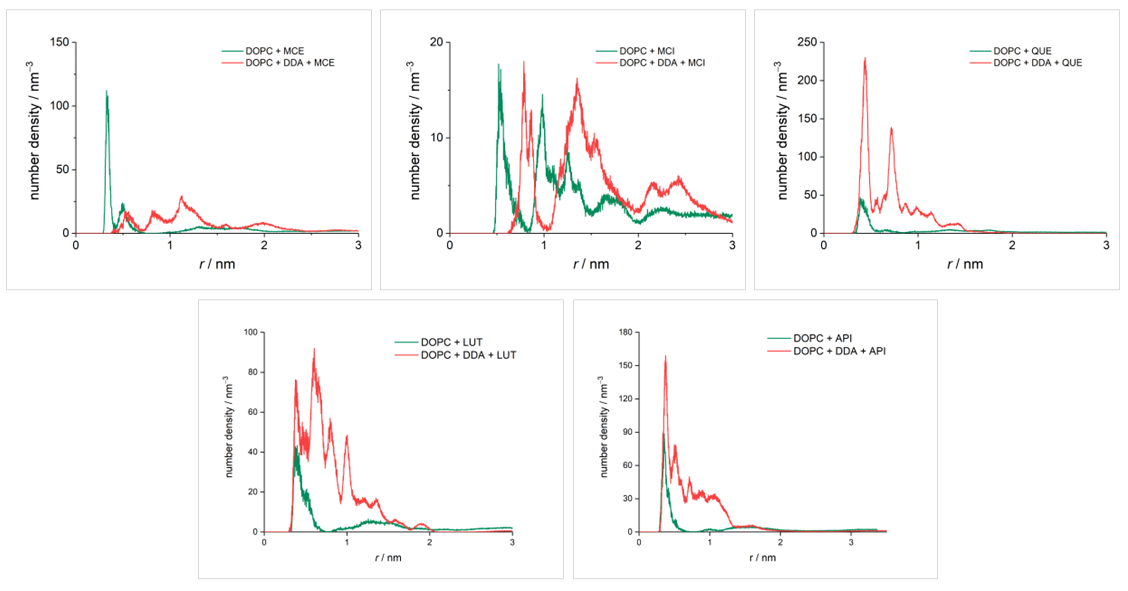


**Figure S3**. Radial distribution functions of flavonoids in the systems with and without DDA.


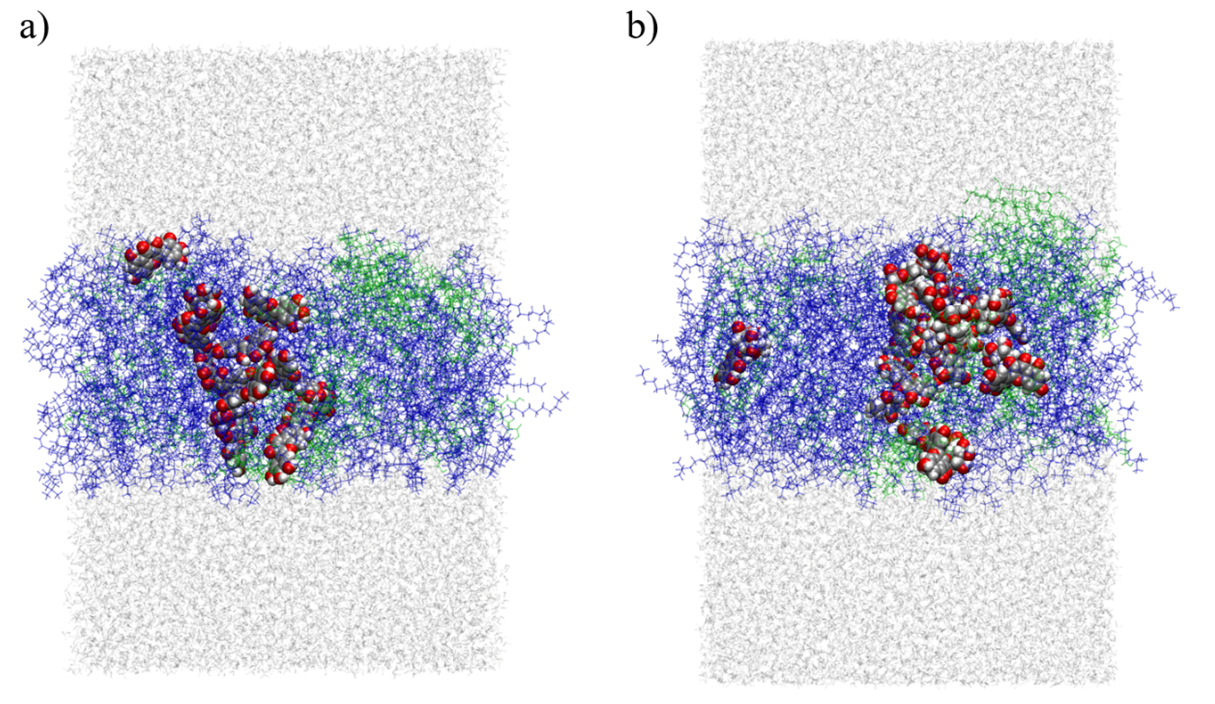


**Figure S4.** Examples of simulation boxes. a) DOPC-DDA with MCE and b) DOPC-DDA with MCI. DOPC is shown in blue, DDA is shown in green, water molecules are shown in light grey, while sphere representation (van der Waals radii) is used to show MCE and MCI. Snapshots of simulation boxes are taken after 10 ns of their respective production runs.

**
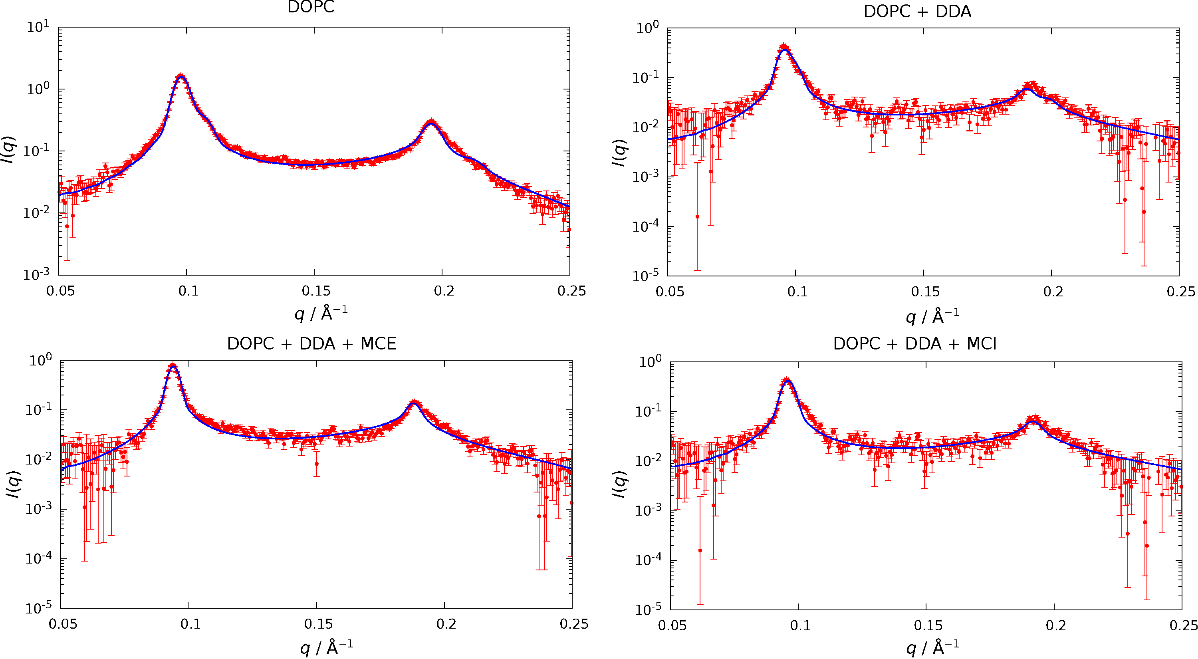
**

**Figure S5.** SAXS curves of the studied DOPC liposomes. Solid blue lines are best-fit curves.

**Table S1**. Parameters obtained from the fittings of experimental SAXS data of multilamellar liposomes.

|  | DOPC | | DOPC + DDA | | DOPC + DDA + MCE | DOPC + DDA + MCI |
| --- | --- | --- | --- | --- | --- | --- |
| w | 0.891 ± 0.002 | 0.109 ± 0.002 | 0.72 ± 0.07 | 0.28 ± 0.07 | 1 | 1 |
| R_pol_ / nm | 0.381 ± 0.009 | | 0.2 ± 0.1 | | 0.35 ± 0.04 | 0.2 ± 0.1 |
| R_CH2_ / nm | 1.03 ± 0.02 | | 1.3 ± 0.4 | | 1.05 ± 0.04 | 1.0 ± 0.2 |
| R_CH3_ /nm | 0.37 ± 0.06 | | 0.28 ± 0.02 | | 0.31 ± 0.03 | 0.34 ± 0.02 |
| R_water_ / nm | 2.71 ± 0.06 | | 2 ± 1 | | 3.5 ± 0.8 | 1.2 ± 1.0 |
| ρ_pol_ / e nm^−3^ | 421 ± 4 | | 380 ± 20 | | 412 ± 6 | 420 ± 22 |
| ρ_CH2_ / e nm^−3^ | 316 ± 3 | | 339 ± 6 | | 320 ± 2 | 320 ± 10 |
| ρ_CH3_ / e nm^−3^ | 243 ± 2 | | 220 ± 12 | | 250 ± 7 | 270 ± 18 |
| ρ_water_ / e nm^−3^ | 330 | | 330 | | 330 | 330 |
| σ_pol_ / nm | 0.27 ± 0.02 | | 0.3 ± 0.2 | | 0.258 ± 0.003 | 0.21 ± 0.08 |
| σ_CH2_ / nm | 0.224 ± 0.005 | | 0.3 ± 0.1 | | 0.3 ± 0.1 | 0.20 ± 0.04 |
| σ_CH3_ / nm | 0.398 ± 0.001 | | 0.5 ± 0.2 | | 0.80 ± 0.06 | 0.7 ± 0.2 |
| N | 12.5 ± 0.4 | | 14 ± 2 | | 16.5 ± 0.7 | 13.9 ± 0.5 |
| d / nm | 6.4048 ± 0.0008 | 5.894 ± 0.008 | 6.59 ± 0.02 | 6.3 ± 0.4 | 6.6771 ± 0.0002 | 6.540 ± 0.005 |
| η_Caillé_ | 0.1070 ± 0.0003 | | 0.124 ± 0.003 | | 0.116 ± 0.001 | 0.128 ± 0.005 |

**Table S2**. Roughness (*R*_a_), thickness (*d*) and Young’s modulus (*E*) of DOPC bilayers before and after the addition of DDA and flavonoids.

|  | DOPC | DOPC + DDA | DOPC + DDA + MCE | DOPC + DDA + MCI |
| --- | --- | --- | --- | --- |
| R_a_ / nm | 0.32 ± 0.08 | 0.46 ± 0.06 | 0.37 ± 0.04 | 0.3 ± 0.1 |
| d / nm | 4.50 ± 0.03 | 4.1 ± 0.1 | 4.4 ± 0.2 | 4.7 ± 0.1 |
| E / MPa | 34.5 ± 1.7 | 21.0 ± 1.6 | 8.0 ± 1.1 | 2.3 ± 0.3 |
